# Volumetric Brain Changes in Older Fallers: A Voxel-Based Morphometric Study

**DOI:** 10.3389/fbioe.2021.610426

**Published:** 2021-03-10

**Authors:** Maxime Le Floch, Pauline Ali, Marine Asfar, Dolores Sánchez-Rodríguez, Mickaël Dinomais, Cédric Annweiler

**Affiliations:** ^1^Department of Geriatric Medicine, Angers University Hospital, Angers University Memory Clinic, Research Center on Autonomy and Longevity, University of Angers, Angers, France; ^2^School of Medicine, Faculty of Health, University of Angers, Angers, France; ^3^Department of Physical and Rehabilitation Medicine, Laboratoire Angevin de Recherche en Ingénierie des Systèmes, Angers University Hospital, Université d’Angers, Angers, France; ^4^Department of Public Health, University of Liège, Liège, Belgium; ^5^Department of Medical Biophysics, Robarts Research Institute, Schulich School of Medicine & Dentistry, University of Western Ontario, London, ON, Canada

**Keywords:** accidental falls, older adults, brain, brain mapping, motor control

## Abstract

**Background:**

Falls are frequent and severe in older adults, especially among those with cognitive impairments due to altered motor control. Which brain areas are affected among fallers remains yet not elucidated. The objective of this cross-sectional analysis was to determine whether the history of falls correlated with focal brain volume reductions in older adults.

**Methods:**

Participants from the MERE study (*n* = 208; mean, 71.9 ± 5.9 years; 43% female; 38% cognitively healthy, 41% with mild cognitive impairment and 21% with dementia) were asked about their history of falls over the preceding year and received a 1.5-Tesla MRI scan of the brain. Cortical gray and white matter subvolumes were automatically segmented using Statistical Parametric Mapping. Age, gender, use of psychoactive drugs, cognitive status, and total intracranial volume were used as covariates.

**Results:**

Fifty-eight participants (28%) reported history of falls. Fallers were older (*P* = 0.001), used more often psychoactive drugs (*P* = 0.008) and had more often dementia (*P* = 0.004) compared to non-fallers. After adjustment, we found correlations between the history of falls and brain subvolumes; fallers exhibiting larger gray matter subvolumes in striatum, principally in bilateral caudate nucleus, than non-fallers. By stratifying on cognitive status, these neuroanatomical correlates were retrieved only in participants with MCI or dementia. There were no correlations with the subvolumes of white matter.

**Conclusion:**

Older fallers had larger subvolumes in bilateral striatum than non-fallers, principally within the caudate nucleus. This suggests a possible brain adaptative mechanism of falls in people with neurocognitive decline.

## Introduction

Falls in older adults are not only frequent, with a prevalence reaching 35% after the age of 65 and 50% after 80, but also severe as they lead to adverse consequences including fractures, hospitalization, loss of independence, institutionalization and death; with significant health care costs (Katz and Shah, 2010). The challenge of the falls is that they can be prevented, at least in part, by identifying and correcting the risk factors for falls (Gillespie et al., 2012). Around 450 risk factors for falls are reported in the literature (Tinetti et al., 1988), including gait and balance disorders among the most important and frequent contributors (Rubenstein et al., 2001).

Human gait is an intentional motor behavior directed toward a goal that ensures the movement of the body in the horizontal plane through postural and balance constraints. Several factors influence motor function and gait performance, whether in the peripheral dimension of motor control (for example the declines in muscle strength, tone or osteoarticular functions) or in its central dimension (essentially the decline of brain health and function) (Allali et al., 2008). Specifically, the brain-level of motor control involves the integration of afferent information in the brain to generate a global motor control message and ultimately to produce complex motor responses that are adapted to multiple sensory inputs and environmental constraints (Annweiler et al., 2013). For this reason, older adults with prodromal or severe cognitive impairments exhibit significant gait disorders (Beauchet et al., 2015). Thus, the efficiency of gait control and the prevention of falls presuppose good health and function of the brain.

Only little is known about the brain changes met among older fallers, the most frequently reported changes being microvascular lesions such as leukoaraiosis (Callisaya et al., 2015). In previous studies, the brain was generally examined over a limited number of regions of interest defined *a priori*, such as the hippocampus and the somatosensory or premotor or prefrontal or parietal cortex, which increases the risk of ignoring unexpected focal changes (Beauchet et al., 2017). In addition, previous brain morphological analyzes have involved specific groups such as Parkinson’s patients, identifying a reduction in gray matter volume (GM) in the right superior temporal gyrus and in the right inferior parietal lobule (Hsu et al., 2016), or within cognitively healthy individuals (CHI) with decreased GM volumes in the orbitofrontal cortex, anterior cingulum, insula, pallidum, and hippocampus (Maidan et al., 2020). To our knowledge, no whole-brain analysis in relation to falls has been conducted yet in individuals with mild cognitive impairment (MCI) or dementia.

We had the opportunity to examine the voxel-based morphometric (VBM) correlations of falls with the whole GM and white matter (WM) volumes in a large representative community survey of older adults with various cognitive statuses in the MERE cohort (Beauchet et al., 2013). We hypothesized that the brain of older fallers would be the seat of specific morphological changes, compared to non-fallers. The objectives of this cross-sectional analysis were (i) to determine whether the history of falls correlated with focal brain volume reductions in the MERE cohort, (ii) to specify the location of these morphological changes, and (iii) to determine if these changes depended on cognitive status.

## Materials and Methods

### Participants

We studied participants followed in the Memory Clinic of the University Hospital of Angers, France, and recruited in the MERE study between November 2009 and 2015 (ClinicalTrials.gov number, NCT01315704). The MERE study is an observational prospective unicentric cohort study designed to examine gait and gait changes with time among older adults visiting the Memory Clinic of Angers University Hospital, France. The sampling and data collection procedures have been described elsewhere in detail (Annweiler et al., 2014a). The main exclusion criteria were age below 60 years, Mini-Mental State Examination (MMSE) score < 10, inability to walk independently, history of stroke, any acute medical illness in the preceding 3 months, current delirium, severe depression defined as 15-item Geriatric Depression Scale score > 10, poor vision, inability to understand or answer the study questionnaires, and refusal to participate in research. All participants included in the present analysis received a full medical examination and a magnetic resonance imaging (MRI) scan of the brain.

### History of Falls

The participants were interviewed using a standardized questionnaire, gathering information on the history of falls over the preceding year. This face-to-face interview was based on standardized questions exploring the number, delay and location of falls (i.e., inside or outside the participant’s house), the evoked causes and circumstances of falls (i.e., syncope or other acute medical event, body transfer from sitting position or walking or other physical activities such as cycling), and all physical traumatisms and inability to get up from the ground after a fall. A fall was defined as an event resulting in a person coming to rest unintentionally on the ground or another lower level, not as the result of a major intrinsic event or an overwhelming hazard. Fallers were defined as having experienced at least one fall in the preceding 12-month period.

### MRI Procedures

#### MRI Acquisition

All images were acquired on the same 1.5 Tesla MRI scanner (Magnetom Avanto, Siemens Medical Solutions, Erlangen, Germany) at the University Hospital of Angers, France, using a standard MRI protocol (Dubois et al., 2009). A high-resolution 3D T1-weighted volume was obtained covering the whole brain (acquisition matrix = 256 × 256 × 144, FOV = 240mm, TE/TR/TI = 4.07 ms/2170 ms/1100 ms, flip angle = 15°, voxel size 1 mm × 1 mm × 1.3 mm).

#### Voxel-Based-Morphometry With DARTEL Analyses

All T1 images were converted from DICOM to NIFTI format using the MRIcron software^1^. Basic voxel-based morphometry (VBM) with DARTEL analysis^2^ was conducted using standard functionalities (default options) available in the VBM8 toolbox^3^ implemented in the SPM8 software^4^. VBM analysis was performed following standard procedures^5^, as previously published (Dinomais et al., 2016). The default options of the VBM procedure provided in VBM8 were used. Native MR images were segmented into distinct tissue classes: GM, WM and cerebrospinal fluid (CSF), using a segmentation approach available in SPM8. The extended option “thorough cleanup,” which is particularly useful for atrophic brain, was used during the first module “estimate and write.” Customized DARTEL-templates were created using affine registered tissue segments (Ashburner, 2007). These customized DARTEL templates replaced the default DARTEL templates. Hence, GM and WM volumes were normalized using high dimensional spatial normalization to a customized DARTEL template. A modulation of the segmented and normalized GM (modulated GM) and WM (modulated WM) volumes were performed (Good et al., 2001). The final resolution of the modulated GM and WM images was 1.5 mm × 1.5 mm × 1.5 mm, but these were smoothed with a 4 mm FWHM (full-width-at-half-maximum) Gaussian Kernel to minimize individual gyral variations. All images were visually inspected to ensure that the steps described above were successful and that each modulated GM and WM map covered the whole brain.

### Covariates

The following variables were used as potential confounders in the analyses: age, gender, use of psychoactive drugs, cognitive status, and total intracranial volume (TIV). Participants were asked to bring all their prescriptions and medications to the clinical center. Psychoactive drugs were defined as benzodiazepines, antidepressants and/or neuroleptics. The cognitive status was diagnosed during multidisciplinary meetings involving geriatricians, neurologists and neuropsychologists of Angers University Memory Center, France, and was based on a variety of standardized neuropsychological tests, physical examination findings, blood tests and MRI brain imaging (Annweiler et al., 2014b). Clinical suspicion of dementia was diagnosed using the Diagnostic and Statistical Manual of Mental Disorders, fourth edition, criteria (Guze, 1995). MCI was diagnosed according to consensual criteria (Albert et al., 2011). Non-demented participants without MCI and who had normal neuropsychological and functional performance were considered as CHI. Finally, the TIV was approximated for each participant by calculating the sum of GM, WM and CSF maps obtained during the pre-processing steps.

### Ethics

Participants were included after having given their informed consent for research. The study was conducted in accordance with the ethical standards set forth in the Helsinki Declaration (1983). The study protocol was approved by the local Ethical Committee (2009/15).

### Statistics

A descriptive analysis of the participants’ characteristics was firstly performed using effectives and frequencies for qualitative variables, and means and standard deviations for continuous variables. Comparisons were performed according to the fallers and non-fallers groups using chi^2^ test or Fisher exact test for qualitative variables, as appropriate, and Student *t*-test or Wilcoxon-Mann-Whitney test for quantitative variables according to the normal distribution assumption.

The smoothed, modulated, normalized imaging datasets were used for voxelwise statistical analysis using SPM8. A whole-brain random-effect full 2 (falls conditions) × 3 (cognitive status) ANOVA was conducted on the MRI data. This statistical analysis allows testing for potential differences between fallers and non-fallers brain volumes and the influence of the cognitive status. Thus, Statistical F-maps were created for each main effect and for each interaction, thus the GM variations across all conditions was determined using an F-contrast. Because F-maps do not contain information about the direction of the main effects, statistical t-contrasts were calculated to determine the direction of any significant main effects. All statistical parametric maps were interpreted after applying a false discovery rate (FDR) correction for multiple comparisons at the whole-brain level with a significance level *p*-Value (corrected) < 0.05. Minimum cluster size was set at 10 contiguous voxels. Anatomic toolbox 2.2c was used for anatomical localizations (Eickhoff et al., 2005). Age, gender, use of psychoactive drugs, cognitive status, and TIV were used as covariates of noninterest implement in our 2X3 full factorial design. Finally, the same analyses were conducted by stratifying on the cognitive status (i.e., within the CHI subgroup (*n* = 79), within the subgroup with MCI (*n* = 86), and finally within the subgroup with dementia (*n* = 43).

## Results

Clinical characteristics and brain subvolumes are presented in Table 1, and further neuropsychological data are provided in Supplementary Appendix 1. The prevalence of falls was 27.8%. Thirty-nine participants reported one fall over the preceding year, while 9 had 2 falls and 10 experienced 3 falls or more. Both groups (i.e., fallers and non-fallers) were similar in terms of total intracranial volume. Fallers were older (*P* = 0.001), more often female (*P* = 0.014), used more often psychoactive drugs (*P* = 0.008) and exhibited more frequent dementia (*P* = 0.004) (Table 1).

**TABLE 1:** Participants’ characteristics (*n* = 208).

	Whole cohort (*n* = 208)	History of falls		*P*-value
		Yes (*n* = 58)	No (*n* = 150)	
Age, years (mean ± SD)	71.9 ± 5.9	74.5 ± 7.0	71 ± 5.0	0.001
Female gender	90 (43.2)	33 (56.9)	57 (38.0)	0.014
Use psychoactive drugs*	49 (23.6)	21 (36.2)	28 (18.7)	0.008
Total intracranial volume, cm^3^ (mean ± SD)	1396.1 ± 127.3	1390.2 ± 140.8	1398.4 ± 122.1	0.676
Cognitive status				0.004
Dementia	43 (20.7)	20 (34.5)	23 (15.3)	
MCI	86 (41.3)	16 (27.6)	70 (46.7)	
Cognitively healthy	79 (38.0)	22 (37.9)	57 (38.0)	

Data presented as n (%) where applicable; MCI: mild cognitive impairment; SD: standard deviation; *Use benzodiazepines and/or antidepressants and/or neuroleptics.

**FIGURE 1:**
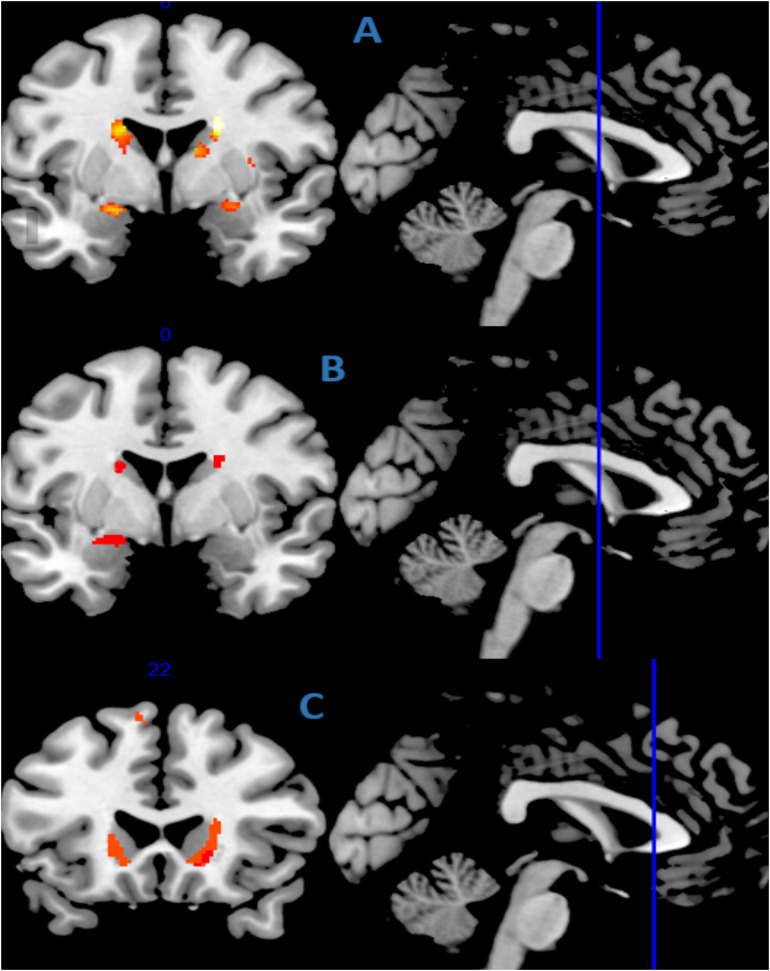
Gray matter regions showing a positive correlation with falls **(A)** in the whole cohort, **(B)** in the subgroup with mild cognitive impairment (*n* = 86), and **(C)** in the subgroup with dementia (*n* = 43), after adjustment for potential confounders (age, gender, use psychoactive drugs, cognitive status, and TIV). The statistical map is co-registered and superimposed on 3D-T1 coronal, axial and sagittal slice (MNI T1 template available on MRICRON software). Results are shown with a significance of *P* < 0.05 and minimum cluster size of 10 continuous voxels.

After adjusting for age, gender, use of psychoactive drugs, cognitive status and TIV, the VBM-DARTEL analysis by using anatomic toolbox 2.2c identified 17 clusters that positively correlated with falls, which are presented in Table 2; the most significant clusters being located in the bilateral caudate nucleus, the bilateral amygdala, the bilateral putamen, the right insula and the left hippocampus. Figure 1A illustrates the main effect of falls and cognitive status on gray matter volumes while adjusting for potential confounders.

**TABLE 2:** Detailed results of the VBM analysis after adjustment for potential confounders: correlation of gray matter volume with falls according to anatomic toolbox 2.2c.

	Brain region	*t-*score	MNI coordinates		
Cluster 1					
(638 voxels)	R Caudate Nucleus	30.36	21	−1	24
	R Caudate Nucleus	28.22	18	24	−5
	R Caudate Nucleus	25.52	20	14	19
	R Caudate Nucleus	21.15	20	−9	19
	R Caudate Nucleus	18.97	12	0	13
Cluster 2					
(513 voxels)	L Caudate Nucleus	27.99	−21	−12	25
	L Caudate Nucleus	24.45	−18	−4	24
	L Caudate Nucleus	19.60	−18	6	21
	L Caudate Nucleus	19.48	−20	11	24
	L Caudate Nucleus	19.39	−21	9	22
	L Caudate Nucleus	16.26	−17	6	12
	L Caudate Nucleus	16.24	−18	5	13
Cluster 3					
(113 voxels)	L Amygdala	20.24	−21	2	−15
Cluster 4					
(109 voxels)	R Putamen	21.78	35	−4	6
	R Putamen	20.46	35	−15	4
	R Putamen	20.12	33	−4	10
	R Insula Lobe	16.43	35	8	15
	R Putamen	15.04	33	2	10
Cluster 5					
(90 voxels)	L Caudate Nucleus	20.00	−17	24	0
	L Caudate Nucleus	18.97	−18	24	4
	L Caudate Nucleus	17.25	−14	23	−8
Cluster 6					
(79 voxels)	R Amygdala	20.62	24	3	−17
	R Amygdala	16.96	27	2	−14
	R Amygdala	15.80	21	−3	−9
	R Amygdala	14.35	23	−1	−11
Cluster 7					
(39 voxels)	L Hippocampus	16.47	−20	−13	−23
Cluster 8					
(34 voxels)	R Precuneus	17.90	8	−45	75
	R Paracentral Lobule	16.13	6	−37	75
Cluster 9					
(20 voxels)	L Insula Lobe	21.58	−30	6	12
Cluster 10					
(19 voxels)	L Posterior-Medial Frontal	19.28	−9	21	58
	L Posterior-Medial Frontal	14.83	−9	17	63
Cluster 11					
(19 voxels)	L Putamen	16.76	−33	−19	1
Cluster 12					
(19 voxels)	R Superial Temporal Gyrus	15.19	42	−4	−15
Cluster 13					
(18 voxels)	L Inferior Temporal Gyrus	15.26	−63	−31	−21
	L Inferior Temporal Gyrus	15.25	−63	−37	−21
Cluster 14					
(17 voxels)	R Globus Pallidum	15.64	11	6	1
Cluster 15					
(15 voxels)	L Middle Temporal Gyrus	15.55	−62	−25	−8
Cluster 16					
(14 voxels)	L Cingular gyrus	16.33	−18	−34	43
Cluster 17					
(13 voxels)	L Middle Temporal Gyrus	18.94	−65	-34	−15

*A threshold of *P* < 0.05, corrected for multiple comparisons based on the false discovery rate (FDR), was applied to the resulting statistical parametric maps. Only clusters with a minimum extent of 10 contiguous voxels are reported. L, Left; R, Right.*

While stratifying on the cognitive status, the history of falls did not correlate with the subvolumes of GM in the CHI subgroup. The subgroup with MCI exhibited correlations with falls mainly in the bilateral caudate nucleus, the left amygdala and the left olfactory cortex after adjusting for potential confounders (Figure 1B) while, in the subgroup with dementia, fallers exhibited a greater GM subvolume principally in the bilateral caudate nucleus, the left middle cingulum and the bilateral insula (Figure 1C). More detailed results and corresponding *t*-tests according to anatomic toolbox 2.2c are shown in Supplementary Appendices 2–6.

Finally, the analysis revealed that falls did not correlate with the subvolumes of white matter across the whole brain.

## Discussion

We found, after adjusting for studied potential confounders, that older fallers had larger gray matter volume in striatum, principally in bilateral caudate nucleus, than non-fallers; a correlation retrieved in those with cognitive decline (either with MCI or dementia). In contrast, there was no between-group difference in white matter subvolumes.

Our study provides evidence of an association between falls and brain subvolumes, notably in the bilateral striatum (i.e., caudate nucleus, putamen). Although the contribution of the striatum to movements is increasingly recognized (Kreitzer and Malenka, 2008), the involvement in falls remains unclear so far. In fact, our results diverge from the hypothesis we originally formulated. We supposed *a priori* that smaller brain subvolumes would be found in fallers since it was previously reported that gait instability, which leads to greater fall risk, may be caused by brain morphological abnormalities such as white matter lesions or gray matter ischemic lesions (Callisaya et al., 2015; Taylor et al., 2018; Blumen et al., 2019). Also, gait disorders were previously associated with focal neuronal loss in brain areas involved in motor skills, visuospatial attention and executive functions (Allali et al., 2014). Briefly, the cerebral motor program is organized into five consecutive steps: sensory feedback, intention, planning, programming and execution (Beauchet et al., 2008), with the striatum being involved in all steps. More precisely, the caudate volume has been directly associated with executive functioning (Macfarlane et al., 2015), which plays a critical role in falls risks among older adults (Muir et al., 2013). Functional imaging data has largely corroborated the model of functional cortico-striatal connectivity (Postuma and Dagher, 2006). This key role of caudate nucleus is highlighted by the facts that Parkinsonian deficits and frontal gait are two main clinical presentations of higher-level gait disorders and are associated with dysfunction in both the basal ganglia and frontal regions (Helmich et al., 2010). Thus, finding morphological changes in caudate nucleus among fallers was not surprising. However, finding an increased subvolume of the caudate nucleus was not expected here, though not unprecedented. For instance, in one recent study, participants with neurosensitive pathology exhibited greater caudate subvolume in response to the lack of environmental information (Casseb et al., 2019). Another study reported that people with schizophrenia had caudate hypertrophy in response to the use of psychoactive drugs (Gur et al., 1998), and similar results were also found in animals models (Andersson et al., 2002).

Enlargement of key regions of the brain could represent an adaptative mechanism to maintain a physiological control of gait. Such brain plasticity corresponds to theories on age-related neurocognitive changes, which support the existence of adaptive strategies to maintain stable brain performance with advancing age (Cabeza et al., 2018). For instance, a recent study showed the effects of normal aging on the neural substrate of gait control using mental imagery during functional MRI of brain: hippocampal regions in older adults exhibited an increased activation compared to younger ones during a task requiring a precise control of gait (i.e., walking on surface consisting of cobbles stones) (Allali et al., 2014), which was interpretated as an adaptative mechanism to maintain a physiological control of gait. Besides normal aging, brain adaptative mechanisms have also been reported in response to functional decline in adults. For example, in functional MRI studies, a greater extent of hippocampal activation has been shown while performing an episodic memory task among patients with early stages of dementia compared to CHI (Dickerson and Sperling, 2008; Smith et al., 2013). This is also consistent with the finding of an increase in caudate volume several years before the clinical onset of familial form of Alzheimer disease (Fortea et al., 2010). Similarly, hypertrophic olivary changes have been reported after axonal damage (Krings et al., 2003). The supplementary recruitment may reflect neurological strategies to cope with structural or functional anomalies (Whalley et al., 2004), even if this adaptation may ultimately be surpassed. Consistently, a J-shaped change in brain subvolumes has been reported while exploring the association between gait variability and brain morphology (Allali et al., 2014; Annweiler et al., 2014b). The increase in MRI subvolumes in the early stages of a pathology may reflect some structural adaptative processes that gradually decrease with the ongoing pathology. Such changes have already been observed in the striatum, more specifically within the bilateral caudate nucleus of patients with long-term neurosensory disorders (Casseb et al., 2019), just like an adaptative mechanism for chronic lack of proprioceptive and vibratory inputs. Since deafferentation increases the risk of falls (Saftari and Kwon, 2018), this may explain at least in part our present results. Of note, we also found here that fallers with MCI and dementia, but not the CHI, exhibited larger GM striatal subvolumes, suggesting an adaptative mechanism used by those with MCI and dementia to maintain operational high-level gait control despite cognitive decline. However, the design of our study did not correlate GM morphological changes to behavioral changes such as gait improvements or prevention of falls, which prevented the conclusion of a compensation mechanism according to Cabeza et al. (2018).

We found no changes in WM subvolumes according to the history of falls. This result is not inconsistent with previous literature supporting a possible contribution of white matter in fall risk, which did not examine white matter volumetry but the onset of white matter hyperintensities (WMH) that broke integrity of white matter fibers (Taylor et al., 2018). It has notably been shown that WMH progression may increase the risk of falls (Callisaya et al., 2015). Thus our findings provide novel information by supporting that the WM might not be responsible for the risk of falling but, if it was the case, it would be a mechanism other than atrophy, such as for example the loss of integrity or vitality of the WM, that would be involved.

To our knowledge, we provide here the first voxel-based morphometric analysis in a large sample of participants to examine the morphology of fallers’ brain. Our sample seems to be representative of the general population of seniors; the clinical characteristics of fallers corresponding to what is known in previous literature and what we observe in clinical routine (No author list, 2001). Regardless, a number of limitations also exist. First, the study sample was restricted to older community-dwellers with cognitive complaints. Second, the search for falls was based on self-report in our patients, which may be influenced by a possible recall bias. Third, our assessment did not include a detailed measure of gait or visual processing, which could have further enhanced our understanding of the underlying mechanisms explaining the association between falls and gray matter subvolumes. Fourth, the cross-sectional design of our study does not allow any causal inference. Fifth, VBM has difficulties with the segmentation and normalization that may result in problems in localization of regional volumes. We limited these defects by using DARTEL, a fluid deformation capable of precisely realigning brain structures (Klein et al., 2009).

In conclusion, we found that older fallers -mostly those with MCI and dementia- had greater subvolumes in bilateral striatum than non-fallers, principally within the caudate nucleus. These findings suggest a possible adaptative mechanism based on the enhancement of specific brain regions to maintain operational high-level gait control despite cognitive decline. Additional work is needed to better understand the neuroanatomical correlates of falls in older adults. Understanding higher-level gait disorders may offer a powerful mechanism to act on mobility decline and falls in older adults and to maintain function late in life.

## Members of the Sam Group

Pierre Abraham, M.D., Ph.D.; Cédric Annweiler, M.D., Ph.D.; Mickael Dinomais, M.D., Ph.D.; Guillaume Duval, M.D., M.S.; Nicolas Lerolle, M.D., Ph.D.; Frédéric Noublanche, M.D., M.S.

## Data Availability Statement

The raw data supporting the conclusions of this article will be made available by the authors, without undue reservation.

## Ethics Statement

The studies involving human participants were reviewed and approved by Ethical Committee of Angers University Hospital. The patients/participants provided their written informed consent to participate in this study.

## Author Contributions

CA had full access to all of the data in the study, took responsibility for the data, the analyses and interpretation and had the right to publish any and all data, separate and apart from the attitudes of the sponsors, performed the administrative, technical, or material support, and supervised the study. CA and ML conceived and designed the study and drafted the manuscript. CA, MA, and ML carried out the data acquisition. ML, PA, MD, and CA did the analysis and interpretation of data. PA, MA, DS-R, and MD critically revised the manuscript for the important intellectual content. PA, MD, and CA did the statistical expertise. All authors have read and approved the manuscript.

## Conflict of Interest

The authors declare that the research was conducted in the absence of any commercial or financial relationships that could be construed as a potential conflict of interest.

## Footnotes

1 https://people.cas.sc.edu/rorden/mricron/index.html

2 http://www.neuro.uni-jena.de/vbm8/VBM8-Manual.pdf

3 http://dbm.neuro.uni-jena.de/VBM8/

4 http://www.fil.ion.ucl.ac.uk/spm

5 http://www.fil.ion.ucl.ac.uk/~john/misc/VBMclass10.pdf
