# Supplementary material for: Volumetric Brain Changes in Older Fallers: A Voxel-Based Morphometric Study
Source: Front Bioeng Biotechnol. 2021 Mar 10;9:610426. doi: 10.3389/fbioe.2021.610426 (PMC7987921; doi:10.3389/fbioe.2021.610426)
Supplement: Supplementary file 1 [file Table_1.docx]

**Appendix 1. Participants’ characteristics according to the cognitive status (n=208)**

|  | **Cognitively healthy (n=79)** | **MCI (n=86)** | **Dementia (n=43)** |
| --- | --- | --- | --- |
| Age, years (mean±SD) | 69.3±3.6 | 70.9±4.8 | 78.6±6 |
| Female gender | 33 (41.7) | 30 (35.8) | 27 (61) |
| Education level |  |  |  |
| Less than primary school | 2 (2.5) | 1 (1.1) | 6 (14) |
| Primary school | 23 (29.1) | 44 (51.1) | 18 (41.9) |
| Secondary school | 28 (35.4) | 28 (32.6) | 12 (28) |
| High school and higher education | 26 (32.9) | 13 (15.1) | 7 (16.3) |
| MMSE score (mean±SD) | 28.4±1.5 | 26.9±2.2 | 20.8±4.3 |
| FAB score (mean±SD) | 16.6±1.6 | 15.7±1.8 | 12±3 |
| Cognitive diagnosis |  |  |  |
| Alzheimer disease | - | - | 35 (81.4) |
| Vascular dementia | - | - | 1 (2.3) |
| Mixed dementia | - | - | 6 (14) |
| Lewy body dementia | - | - | 1 (2.3) |

Data presented as n (%) where applicable; FAB: Frontal Assessment Battery; MCI: mild cognitive impairment; MMSE: Mini-Mental State Examination; SD: standard deviation
