# Supplementary material for: Volumetric Brain Changes in Older Fallers: A Voxel-Based Morphometric Study
Source: Front Bioeng Biotechnol. 2021 Mar 10;9:610426. doi: 10.3389/fbioe.2021.610426 (PMC7987921; doi:10.3389/fbioe.2021.610426)
Supplement: Supplementary file 2 [file Table_2.docx]

**Appendix 2**. **Detailed results of the VBM analysis according to anatomic toolbox2.2c after adjustment for potential confounders: *t*-test corresponding to the hypothesis that fallers exhibited greater gray matter subvolumes than non-fallers. A threshold of P<0.05, corrected for multiple comparisons based on the false discovery rate (FDR), was applied to the resulting statistical parametric maps. Only clusters with a minimum extent of 10 contiguous voxels are reported.**

|  | **Brain region** | ***t-*score** | **MNI coordinates** | | |
| --- | --- | --- | --- | --- | --- |
| Cluster 1 (**799 vox**) |  |  |  |  |  |
|  | R Caudate Nucleus | 5.51 | 21 | -1 | 24 |
|  | R Caudate Nucleus | 5.31 | 18 | 24 | -5 |
|  | R Caudate Nucleus | 5.05 | 20 | 14 | 19 |
|  | R Caudate Nucleus | 4.60 | 20 | -9 | 19 |
|  | R Caudate Nucleus | 4.36 | 12 | 0 | 13 |
|  | R Caudate Nucleus | 3.96 | 11 | 6 | 1 |
| Cluster 2 (**679 vox**) |  |  |  |  |  |
|  | L Caudate Nucleus | 5.29 | -21 | -12 | 25 |
|  | L Caudate Nucleus | 4.94 | -18 | -4 | 24 |
|  | L Caudate Nucleus | 4.43 | -18 | 6 | 21 |
|  | L Caudate Nucleus | 4.41 | -20 | 11 | 24 |
|  | L Caudate Nucleus | 4.40 | -21 | 9 | 22 |
|  | L Caudate Nucleus | 4.03 | -17 | 6 | 12 |
|  | L Caudate Nucleus | 4.03 | -18 | 5 | 13 |
|  | L Caudate Nucleus | 3.33 | -20 | 21 | 12 |
| Cluster 3 (**469 vox**) |  |  |  |  |  |
|  | L Amygdala | 4.50 | -21 | 2 | -15 |
|  | L ParaHippocampal Gyrus | 4.11 | -18 | -19 | -27 |
|  | L Hippocampus | 4.06 | -20 | -13 | -23 |
|  | L Hippocampus | 3.77 | -24 | -12 | -15 |
|  | L ParaHippocampal Gyrus | 3.48 | -24 | -21 | -24 |
| Cluster 4 (**259 vox**) |  |  |  |  |  |
|  | R Putamen | 4.67 | 35 | -4 | 6 |
|  | R Putamen | 4.52 | 35 | -15 | 4 |
|  | R Putamen | 4.49 | 33 | -4 | 10 |
|  | R Insula Lobe | 4.05 | 35 | 8 | 15 |
|  | R Insula Lobe | 3.90 | 41 | -13 | 4 |
|  | R Putamen | 3.88 | 33 | 2 | 10 |
|  | R Insula Lobe | 3.61 | 36 | 3 | 16 |
|  | R Insula Lobe | 3.58 | 35 | 0 | 16 |
| Cluster 5 (**179 vox**) |  |  |  |  |  |
|  | R Amygdala | 4.54 | 24 | 3 | -17 |
|  | R Amygdala | 4.12 | 27 | 2 | -14 |
|  | R Amygdala | 3.98 | 21 | -3 | -9 |
|  | R Amygdala | 3.79 | 23 | -1 | -11 |
| Cluster 6 (**131 vox**) |  |  |  |  |  |
|  | L Caudate Nucleus | 4.47 | -17 | 24 | 0 |
|  | L Caudate Nucleus | 4.36 | -18 | 24 | 4 |
|  | L Caudate Nucleus | 4.15 | -14 | 23 | -8 |
| Cluster 7 (**100 vox**) |  |  |  |  |  |
|  | L Middle Temporal Gyrus | 4.35 | -65 | -34 | -15 |
|  | L Inferior Temporal Gyrus | 3.91 | -63 | -31 | -21 |
|  | L Inferior Temporal Gyrus | 3.91 | -63 | -37 | -21 |
| Cluster 8 (**79 vox**) |  |  |  |  |  |
|  | L Putamen | 4.09 | -33 | -19 | 1 |
| Cluster 9 (**68 vox**) |  |  |  |  |  |
|  | L Cingular Gyrus | 4.04 | -18 | -34 | 43 |
|  | L Cingular Gyrus | 3.65 | -14 | -30 | 37 |
| Cluster 10 (**66 vox**) |  |  |  |  |  |
|  | L Posterior-Medial Frontal | 4.39 | -9 | 21 | 58 |
|  | L Posterior-Medial Frontal | 3.85 | -9 | 17 | 63 |
| Cluster 11 **(59 vox**) |  |  |  |  |  |
|  | R Precuneus | 4.23 | 8 | -45 | 75 |
|  | R Paracentral Lobule | 4.02 | 6 | -37 | 75 |
| Cluster 12 (**51 vox**) |  |  |  |  |  |
|  | R Superior Temporal Gyrus | 3.90 | 42 | -4 | -15 |
| Cluster 13 (**49 vox**) |  |  |  |  |  |
|  | L Insula Lobe | 4.65 | -30 | 6 | 12 |
|  | L Rolandic Operculum | 4.29 | -38 | 3 | 16 |
|  | L Insula Lobe | 3.96 | -36 | 0 | 18 |
| Cluster 14 (**41 vox**) |  |  |  |  |  |
|  | L Putamen | 3.74 | -33 | -4 | 1 |
| Cluster 15 (**38 vox**) |  |  |  |  |  |
|  | L Fusiform Gyrus | 3.67 | -29 | -30 | -21 |
| Cluster 16 (**36 vox**) |  |  |  |  |  |
|  | L Pallidum | 3.80 | -11 | 6 | -2 |
| Cluster 17 (**34 vox**) |  |  |  |  |  |
|  | L Middle Temporal Gyrus | 3.94 | -62 | -25 | -8 |
| Cluster 18 (**28 vox**) |  |  |  |  |  |
|  | L Insula Lobe | 4.03 | -33 | -7 | 12 |
|  | L Insula Lobe | 3.41 | -38 | -12 | 12 |
| Cluster 19 (**26 vox**) |  |  |  |  |  |
|  | R Fusiform Gyrus | 3.53 | 32 | -37 | -15 |
| Cluster 20 (**22 vox**) |  |  |  |  |  |
|  | L Fusiform Gyrus | 4.07 | -48 | -60 | -20 |
| Cluster 21 (**16 vox**) |  |  |  |  |  |
|  | R ParaHippocampal Gyrus | 4.01 | 21 | -21 | -18 |
| Cluster 22 **(15 vox**) |  |  |  |  |  |
|  | Hypophysis | 3.87 | 2 | -6 | -12 |
| Cluster 23 (**14 vox**) |  |  |  |  |  |
|  | L Temporal Pole | 4.03 | -44 | 3 | -17 |
| Cluster 24 (**12 vox**) |  |  |  |  |  |
|  | L Precuneus | 4.19 | -11 | -55 | 43 |
| Cluster 25 (**12 vox**) |  |  |  |  |  |
|  | R Temporal Pole | 3.84 | 32 | 8 | -21 |
