# Supplementary material for: Volumetric Brain Changes in Older Fallers: A Voxel-Based Morphometric Study
Source: Front Bioeng Biotechnol. 2021 Mar 10;9:610426. doi: 10.3389/fbioe.2021.610426 (PMC7987921; doi:10.3389/fbioe.2021.610426)
Supplement: Supplementary file 3 [file Table_3.docx]

**Appendix 3. Detailed results of VBM analysis according to anatomic toolbox2.2c after adjustment for potential confounders: main effect of cognitive status on gray matter subvolumes. A threshold of P<0.05, corrected for multiple comparisons based on the false discovery rate (FDR), was applied to the resulting statistical parametric maps. Only clusters with a minimum extent of 10 contiguous voxels are reported.**

|  | **Brain region** | ***t-*score** | **MNI coordinates** | | |
| --- | --- | --- | --- | --- | --- |
| Cluster 1 (**19256 vox**) |  |  |  |  |  |
|  | L Hippocampus | 28.91 | -26 | -9 | -17 |
|  | R Hippocampus | 25.59 | 26 | -9 | -15 |
|  | L Hippocampus | 24.94 | -20 | -3 | -23 |
|  | L Middle Temporal Gyrus | 20.08 | -59 | -30 | -5 |
|  | L Putamen | 19.83 | -14 | 3 | -11 |
|  | L Putamen | 19.18 | -9 | 8 | -9 |
|  | L Hippocampus | 18.34 | -30 | -16 | -14 |
|  | L Fusiform Gyrus | 17.90 | -30 | -37 | -20 |
|  | L Lingual Gyrus | 17.36 | -17 | -39 | -2 |
|  | L Fusiform Gyrus | 16.55 | -30 | -28 | -24 |
|  | L Insula Lobe | 16.16 | -36 | 21 | 6 |
| Cluster 2 (**2253 vox**) |  |  |  |  |  |
|  | R Middle Temporal Gyrus | 14.34 | 59 | -40 | 3 |
|  | R Superior Temporal Gyrus | 13.61 | 54 | -27 | -2 |
|  | R Superior Temporal Gyrus | 13.45 | 57 | -25 | -2 |
|  | R Middle Temporal Gyrus | 12.47 | 53 | -31 | 0 |
|  | R Inferior Temporal Gyrus | 11.74 | 60 | -9 | -29 |
|  | R Inferior Temporal Gyrus | 11.34 | 62 | -8 | -27 |
|  | R Middle Temporal Gyrus | 11.18 | 54 | -24 | -9 |
|  | R Middle Temporal Gyrus | 10.63 | 51 | -31 | -6 |
|  | R Middle Temporal Gyrus | 10.42 | 66 | -15 | -21 |
|  | R Superior Temporal Gyrus | 9.20 | 57 | -31 | 3 |
|  | R Middle Temporal Gyrus | 9.07 | 66 | -46 | -3 |
| Cluster 3 (**1250 vox**) |  |  |  |  |  |
|  | R PCC | 12.19 | 5 | -43 | 22 |
|  | R PCC | 11.07 | 5 | -39 | 28 |
|  | L PCC | 9.80 | -3 | -43 | 22 |
|  | L PCC | 9.61 | -3 | -43 | 25 |
|  | L Precuneus | 8.78 | -14 | -46 | 39 |
|  | L MCC | 8.43 | -3 | -27 | 33 |
|  | L PCC | 8.24 | -5 | -48 | 33 |
|  | L PCC | 7.75 | -3 | -36 | 30 |
|  | R MCC | 7.62 | 5 | -27 | 33 |
|  | L PCC | 7.36 | -3 | -49 | 25 |
|  | L MCC | 6.97 | -2 | -30 | 40 |
| Cluster 4 (**1114 vox**) |  |  |  |  |  |
|  | L Inferior Parietal Lobule | 10.22 | -50 | -42 | 48 |
|  | L Angular Gyrus | 9.46 | -48 | -66 | 39 |
|  | L Angular Gyrus | 9.38 | -41 | -69 | 49 |
|  | L Angular Gyrus | 9.34 | -45 | -63 | 43 |
|  | L Inferior Parietal Lobule | 9.14 | -36 | -58 | 51 |
|  | L SupraMarginal Gyrus | 9.03 | -57 | -33 | 37 |
|  | L Inferior Parietal Lobule | 8.87 | -48 | -52 | 49 |
|  | L Angular Gyrus | 8.66 | -47 | -70 | 28 |
|  | L Inferior Parietal Lobule | 8.38 | -42 | -60 | 51 |
|  | L Middle Temporal Gyrus | 8.29 | -45 | -75 | 18 |
|  | L Inferior Parietal Lobule | 8.17 | -42 | -40 | 43 |
| Cluster 5 (**712 vox**) |  |  |  |  |  |
|  | R Superior Temporal Gyrus | 11.39 | 53 | -7 | 0 |
|  | R Amygdala | 8.89 | 36 | -10 | -9 |
|  | R Insula Lobe | 8.70 | 36 | -18 | 15 |
|  | R Insula Lobe | 8.27 | 38 | -15 | 0 |
|  | R Amydala | 8.07 | 36 | -6 | -9 |
|  | R Heschls Gyrus | 7.82 | 48 | -15 | 4 |
|  | R Insula Lobe | 7.78 | 36 | -15 | 9 |
|  | R Superior Temporal Gyrus | 7.41 | 48 | -9 | -9 |
|  | R Insula Lobe | 7.36 | 42 | -13 | 9 |
|  | R Insula Lobe | 6.27 | 41 | 2 | -9 |
| Cluster 6 (**692 vox**) |  |  |  |  |  |
|  | R Inferior Parietal Lobule | 12.15 | 56 | -34 | 52 |
|  | R Postcentral Gyrus | 8.61 | 57 | -15 | 43 |
|  | R Postcentral Gyrus | 7.48 | 44 | -27 | 49 |
|  | R Postcentral Gyrus | 7.38 | 57 | -18 | 46 |
|  | R Postcentral Gyrus | 7.33 | 60 | -12 | 33 |
|  | R SupraMarginal Gyrus | 7.15 | 56 | -25 | 40 |
|  | R Postcentral Gyrus | 6.91 | 65 | -13 | 21 |
|  | R Postcentral Gyrus | 6.79 | 62 | -12 | 28 |
|  | R SupraMarginal Gyrus | 6.65 | 60 | -36 | 43 |
|  | RPrecentral Gyrus | 6.62 | 50 | -9 | 48 |
|  | R Postcentral Gyrus | 6.53 | 54 | -3 | 27 |
| Cluster 7 (**673 vox**) |  |  |  |  |  |
|  | L Cerebelum (IX) | 8.84 | -6 | -52 | -57 |
|  | L Cerebelum (IX) | 8.74 | -9 | -46 | -53 |
|  | L Cerebelum (IX) | 8.72 | -9 | -46 | -60 |
|  | R Cerebelum (IX) | 8.72 | 2 | -55 | -45 |
|  | L Cerebelum (IX) | 7.30 | -2 | -54 | -53 |
|  | R Cerebelum (IX) | 6.82 | 9 | -51 | -51 |
|  | L Cerebelum (IX) | 5.39 | -17 | -43 | -54 |
| Cluster 8 (**540 vox**) |  |  |  |  |  |
|  | L Inferior Temporal Gyrus | 11.32 | -57 | -40 | -26 |
|  | L Fusiform Gyrus | 9.86 | -42 | -49 | -14 |
|  | L Inferior Temporal Gyrus | 9.82 | -47 | -54 | -12 |
|  | L Inferior Temporal Gyrus | 8.39 | -47 | -46 | -18 |
|  | L Inferior Occipital Gyrus | 7.95 | -41 | -64 | -8 |
|  | L Inferior Temporal Gyrus | 7.73 | -48 | -40 | -26 |
|  | L Fusiform Gyrus | 7.15 | -42 | -43 | -23 |
|  | L Inferior Occipital Gyrus | 7.08 | -47 | -60 | -11 |
|  | L Inferior Temporal Gyrus | 5.72 | -45 | -36 | -24 |
| Cluster 9 (**487 vox**) |  |  |  |  |  |
|  | L Insula Lobe | 10.69 | -32 | -21 | 18 |
|  | L Rolandic Operculum | 7.90 | -39 | -25 | 22 |
|  | L Insula Lobe | 7.69 | -39 | -7 | 7 |
|  | L Insula Lobe | 7.52 | -38 | -10 | 12 |
|  | L Rolandic Operculum | 7.13 | -39 | -31 | 18 |
|  | L Superior Temporal Gyrus | 7.09 | -47 | -33 | 16 |
|  | L Rolandic Operculum | 6.82 | -45 | -19 | 16 |
|  | L Heschls Gyrus | 6.47 | -38 | -21 | 7 |
|  | L Heschls Gyrus | 6.46 | -38 | -24 | 10 |
|  | L Heschls Gyrus | 6.29 | -36 | -27 | 12 |
|  | L Heschls Gyrus | 5.66 | -42 | -16 | 9 |
| Cluster 10 (**476 vox**) |  |  |  |  |  |
|  | R Superior Orbital Gyrus | 10.31 | 15 | 63 | -12 |
|  | R Middle Orbital Gyrus | 9.97 | 33 | 53 | -14 |
|  | R Middle Orbital Gyrus | 8.32 | 36 | 59 | -12 |
|  | R Middle Orbital Gyrus | 8.31 | 27 | 54 | -17 |
|  | R Middle Orbital Gyrus | 8.21 | 26 | 56 | -15 |
|  | R Superior Orbital Gyrus | 7.69 | 27 | 57 | -3 |
|  | R Middle Orbital Gyrus | 7.46 | 39 | 48 | -18 |
|  | R Superior Orbital Gyrus | 7.45 | 11 | 59 | -18 |
|  | R Superior Orbital Gyrus | 6.72 | 26 | 60 | -6 |
|  | R Superior Orbital Gyrus | 6.42 | 21 | 59 | -3 |
|  | R Middle Orbital Gyrus | 6.42 | 41 | 53 | -15 |
| Cluster 11 (**412 vox**) |  |  |  |  |  |
|  | L Postcentral Gyrus | 9.63 | -51 | -13 | 39 |
|  | L Postcentral Gyrus | 8.52 | -53 | -10 | 46 |
|  | L Postcentral Gyrus | 7.77 | -54 | -10 | 31 |
|  | L Postcentral Gyrus | 7.56 | -48 | -9 | 28 |
|  | L Precentral Gyrus | 6.47 | -51 | -4 | 30 |
|  | L Precentral Gyrus | 6.36 | -54 | -3 | 30 |
|  | L Postcentral Gyrus | 6.30 | -54 | -7 | 40 |
|  | L Postcentral Gyrus | 6.11 | -53 | -6 | 42 |
|  | L Precentral Gyrus | 6.04 | -54 | -4 | 36 |
| Cluster 12 (**343 vox**) |  |  |  |  |  |
|  | R Middle Frontal Gyrus | 11.76 | 42 | 53 | 18 |
|  | R Middle Frontal Gyrus | 9.78 | 39 | 57 | 1 |
|  | R Middle Frontal Gyrus | 8.39 | 33 | 57 | 15 |
|  | R Middle Frontal Gyrus | 8.38 | 39 | 56 | 13 |
|  | R Middle Frontal Gyrus | 8.20 | 29 | 59 | 6 |
|  | R Middle Frontal Gyrus | 7.80 | 38 | 54 | 6 |
|  | R Superior Frontal Gyrus | 7.80 | 21 | 63 | 6 |
|  | R Superior Medial Gyrus | 6.44 | 15 | 63 | 4 |
|  | R Middle Frontal Gyrus | 6.43 | 41 | 48 | 10 |
|  | R Middle Frontal Gyrus | 5.75 | 47 | 47 | 19 |
| Cluster 13 (**337 vox**) |  |  |  |  |  |
|  | L Superior Orbital Gyrus | 9.97 | -24 | 57 | -6 |
|  | L Middle Frontal Gyrus | 9.30 | -38 | 53 | 18 |
|  | L Middle Frontal Gyrus | 8.86 | -27 | 56 | 10 |
|  | L Superior Frontal Gyrus | 8.61 | -23 | 63 | 1 |
|  | L Middle Frontal Gyrus | 8.42 | -29 | 51 | 15 |
|  | L Superior Orbital Gyrus | 5.66 | -20 | 63 | -3 |
|  | L Superior Frontal Gyrus | 5.26 | -21 | 57 | 3 |
| Cluster 14 (**278 vox**) |  |  |  |  |  |
|  | R Insula Lobe | 14.61 | 41 | 18 | 4 |
|  | R Insula Lobe | 6.61 | 33 | 15 | 6 |
| Cluster 15 (**276 vox**) |  |  |  |  |  |
|  | R Middle Occipital Gyrus | 10.38 | 38 | -82 | 10 |
|  | R Middle Occipital Gyrus | 10.35 | 45 | -73 | 18 |
|  | R Middle Temporal Gyrus | 9.98 | 48 | -75 | 12 |
|  | R Middle Occipital Gyrus | 9.50 | 36 | -85 | 6 |
|  | R Middle Temporal Gyrus | 5.53 | 53 | -72 | 6 |
| Cluster 16 (**256 vox**) |  |  |  |  |  |
|  | R Middle Orbital Gyrus | 14.74 | 27 | 38 | -14 |
|  | R IFG (p. Orbitalis) | 11.18 | 29 | 33 | -17 |
|  | R IFG (p. Orbitalis) | 8.09 | 33 | 36 | -11 |
| Cluster 17 (**225 vox**) |  |  |  |  |  |
|  | L Middle Frontal Gyrus | 10.38 | -41 | 50 | 0 |
|  | L Middle Frontal Gyrus | 8.94 | -44 | 50 | 4 |
|  | L IFG (p. Triangularis) | 8.36 | -48 | 42 | 1 |
|  | L IFG (p. Orbitalis) | 6.24 | -45 | 42 | -8 |
|  | L Middle Frontal Gyrus | 5.70 | -36 | 56 | 4 |
| Cluster 18 (**193 vox**) |  |  |  |  |  |
|  | R Superior Temporal Gyrus | 12.98 | 51 | -28 | 12 |
|  | R Superior Temporal Gyrus | 6.59 | 68 | -24 | 9 |
| Cluster 19 (**160 vox**) |  |  |  |  |  |
|  | R Insula Lobe | 7.26 | 35 | 17 | -11 |
|  | R Insula Lobe | 7.11 | 36 | 23 | -3 |
|  | R Insula Lobe | 6.87 | 35 | 26 | 0 |
|  | R IFG (p. Orbitalis) | 6.42 | 33 | 27 | -5 |
| Cluster 20 (**150 vox**) |  |  |  |  |  |
|  | R Lingual Gyrus | 11.16 | 21 | -76 | -12 |
|  | R Fusiform Gyrus | 9.71 | 26 | -81 | -15 |
|  | R Lingual Gyrus | 7.54 | 12 | -81 | -14 |
|  | R Lingual Gyrus | 5.85 | 8 | -78 | -11 |
| Cluster 21 (**149 vox**) |  |  |  |  |  |
|  | L Postcentral Gyrus | 8.98 | -47 | -22 | 58 |
|  | L Postcentral Gyrus | 8.38 | -36 | -30 | 69 |
|  | L Postcentral Gyrus | 6.91 | -50 | -19 | 48 |
|  | L Postcentral Gyrus | 6.78 | -39 | -28 | 64 |
| Cluster 22 (**137 vox**) |  |  |  |  |  |
|  | R Calcarine Gyrus | 9.11 | 9 | -61 | 12 |
|  | R Calcarine Gyrus | 8.62 | 3 | -64 | 18 |
|  | R Precuneus | 6.10 | 8 | -55 | 18 |
| Cluster 23 (**129 vox**) |  |  |  |  |  |
|  | R Superior Parietal Lobule | 8.58 | 29 | -61 | 52 |
|  | R Superior Occipital Gyrus | 7.68 | 26 | -73 | 45 |
|  | R Superior Parietal Lobule | 7.38 | 26 | -69 | 48 |
|  | R Superior Occipital Gyrus | 7.26 | 27 | -76 | 40 |
|  | R Superior Parietal Lobule | 5.99 | 30 | -66 | 51 |
| Cluster 24 (**127 vox**) |  |  |  |  |  |
|  | R Inferior Temporal Gyrus | 10.73 | 53 | -70 | -5 |
|  | R Inferior Occipital Gyrus | 6.09 | 48 | -76 | -6 |
|  | R Inferior Temporal Gyrus | 5.95 | 48 | -67 | -12 |
| Cluster 25 (**126 vox)** |  |  |  |  |  |
|  | L Middle Frontal Gyrus | 8.30 | -24 | 20 | 48 |
|  | L Middle Frontal Gyrus | 7.72 | -32 | 12 | 52 |
|  | L Middle Frontal Gyrus | 7.17 | -21 | 26 | 46 |
|  | L Superior Frontal Gyrus | 5.53 | -20 | 30 | 48 |
| Cluster 26 (**118 vox**) |  |  |  |  |  |
|  | L Middle Frontal Gyrus | 13.25 | -35 | 41 | 36 |
| Cluster 27 (**113 vox**) |  |  |  |  |  |
|  | R Superior Frontal Gyrus | 7.82 | 26 | 6 | 58 |
|  | R Superior Frontal Gyrus | 7.51 | 24 | -6 | 57 |
|  | R Superior Frontal Gyrus | 6.25 | 26 | -1 | 61 |
| Cluster 28 (**109 vox**) |  |  |  |  |  |
|  | R Middle Frontal Gyrus | 7.06 | 29 | 33 | 45 |
|  | R Middle Frontal Gyrus | 6.87 | 36 | 32 | 43 |
|  | R Middle Frontal Gyrus | 6.45 | 23 | 29 | 43 |
|  | R Middle Frontal Gyrus | 5.97 | 29 | 24 | 45 |
| Cluster 29 (**104 vox**) |  |  |  |  |  |
|  | L MCC | 9.86 | -8 | 6 | 43 |
|  | L MCC | 7.19 | -6 | 12 | 40 |
| Cluster 30 (**94 vox**) |  |  |  |  |  |
|  | R Rolandic Operculum | 8.17 | 44 | -27 | 22 |
|  | R Rolandic Operculum | 7.90 | 44 | -24 | 18 |
|  | R Rolandic Operculum | 7.78 | 41 | -25 | 19 |
| Cluster 31 (**94 vox**) |  |  |  |  |  |
|  | R Inferior Temporal Gyrus | 10.36 | 59 | -61 | -12 |
|  | R Inferior Temporal Gyrus | 5.95 | 62 | -58 | -5 |
| Cluster 32 (**93 vox**) |  |  |  |  |  |
|  | Cerebellar Vermis (4/5) | 8.29 | 0 | -61 | -8 |
| Cluster 33 (**86 vox**) |  |  |  |  |  |
|  | R IFG (p. Triangularis) | 7.69 | 54 | 29 | 12 |
|  | R IFG (p. Triangularis) | 7.24 | 51 | 35 | 15 |
|  | R IFG (p. Triangularis) | 6.81 | 54 | 39 | 6 |
| Cluster 34 (**73 vox**) |  |  |  |  |  |
|  | R Inferior Temporal Gyrus | 8.73 | 48 | -52 | -12 |
| Cluster 35 (**70 vox**) |  |  |  |  |  |
|  | R Rolandic Operculum | 7.99 | 50 | 5 | 0 |
|  | R Rolandic Operculum | 6.46 | 53 | 2 | 4 |
| Cluster 36 (**63 vox**) |  |  |  |  |  |
|  | R Middle Occipital Gyrus | 9.07 | 35 | -78 | 25 |
|  | R Middle Occipital Gyrus | 6.81 | 33 | -78 | 33 |
|  | R Middle Occipital Gyrus | 6.03 | 32 | -75 | 39 |
| Cluster 37 (**63 vox**) |  |  |  |  |  |
|  | L Caudate Nucleus | 9.21 | -3 | 18 | 1 |
|  | L Olfactory cortex | 5.92 | -5 | 23 | -2 |
| Cluster 38 (**60 vox**) |  |  |  |  |  |
|  | L Superior Medial Gyrus | 6.77 | -11 | 59 | 16 |
|  | L Superior Frontal Gyrus | 6.44 | -15 | 68 | 9 |
|  | L Superior Medial Gyrus | 5.30 | -6 | 60 | 9 |
| Cluster 39 (56 vox) |  |  |  |  |  |
|  | L Fusiform Gyrus | 7.88 | -39 | -55 | -21 |
|  | L Fusiform Gyrus | 7.26 | -38 | -64 | -17 |
| Cluster 40 (**54 vox**) |  |  |  |  |  |
|  | L Superior Parietal Lobule | 10.24 | -23 | -48 | 70 |
|  | L Precuneus | 6.35 | -17 | -45 | 69 |
| Cluster 41 (**52 vox)** |  |  |  |  |  |
|  | R Cerebelum (Crus 1) | 10.56 | 36 | -67 | -33 |
| Cluster 42 (**51 vox**) |  |  |  |  |  |
|  | R Posterior-Medial Frontal | 9.86 | 9 | 2 | 51 |
|  | R Posterior-Medial Frontal | 7.35 | 9 | -6 | 48 |
| Cluster 43 **(50 vox**) |  |  |  |  |  |
|  | L Middle Occipital Gyrus | 7.94 | -45 | -73 | 0 |
|  | L Inferior Occipital Gyrus | 5.88 | -50 | -75 | -5 |
| Cluster 44 (**48 vox**) |  |  |  |  |  |
|  | L Middle Temporal Gyrus | 7.19 | -60 | -18 | -17 |
|  | L Middle Temporal Gyrus | 6.34 | -68 | -13 | -17 |
|  | L Middle Temporal Gyrus | 6.19 | -65 | -15 | -15 |
| Cluster 45 (**41 vox**) |  |  |  |  |  |
|  | R Superior Frontal Gyrus | 6.25 | 23 | 21 | 46 |
| Cluster 46 (**38 vox**) |  |  |  |  |  |
|  | L Posterior-Medial Frontal | 8.33 | -5 | -10 | 52 |
|  | L Posterior-Medial Frontal | 6.20 | -2 | -12 | 55 |
| Cluster 47 (**33 vox**) |  |  |  |  |  |
|  | L SupraMarginal Gyrus | 7.84 | -54 | -42 | 31 |
| Cluster 48 (**29 vox**) |  |  |  |  |  |
|  | R IFG (p. Opercularis) | 6.34 | 54 | 11 | 21 |
|  | R IFG (p. Opercularis) | 5.50 | 53 | 9 | 27 |
| Cluster 49 (**29 vox**) |  |  |  |  |  |
|  | R Inferior Temporal Gyrus | 6.30 | 53 | -26 | -29 |
|  | R Inferior Temporal Gyrus | 5.86 | 50 | -21 | -27 |
| Cluster 50 (**28 vox**) |  |  |  |  |  |
|  | R Caudate nucleus | 7.85 | 14 | 0 | 9 |
|  | R Caudate nucleus | 5.84 | 12 | 3 | 3 |
| Cluster 51 (**28 vox**) |  |  |  |  |  |
|  | L Medial Temporal Pole | 6.46 | -24 | 17 | -36 |
| Cluster 52 (**27 vox)** |  |  |  |  |  |
|  | L Middle Frontal Gyrus | 7.50 | -27 | 35 | 40 |
| Cluster 53 (**26 vox**) |  |  |  |  |  |
|  | R ACC | 6.74 | 2 | 35 | 19 |
| Cluster 54 (**25 vox**) |  |  |  |  |  |
|  | R SupraMarginal Gyrus | 6.41 | 59 | -46 | 36 |
| Cluster 55 (**25 vox**) |  |  |  |  |  |
|  | R Fusiform Gyrus | 8.21 | 39 | -36 | -27 |
| Cluster 56 (**25 vox**) |  |  |  |  |  |
|  | R Fusiform Gyrus | 7.15 | 38 | -18 | -30 |
| Cluster 57 (**24 vox**) |  |  |  |  |  |
|  | L Superior Parietal Lobule | 6.66 | -30 | -48 | 66 |
|  | L Superior Parietal Lobule | 6.66 | -30 | -52 | 66 |
| Cluster 58 (**24 vox**) |  |  |  |  |  |
|  | L Postcentral Gyrus | 6.24 | -41 | -28 | 45 |
|  | L Inferior Parietal Lobule | 6.12 | -44 | -27 | 40 |
| Cluster 59 (**24 vox**) |  |  |  |  |  |
|  | R Lingual Gyrus | 7.16 | 11 | -54 | 6 |
| Cluster 60 (**24 vox**) |  |  |  |  |  |
|  | R Medial Temporal Pole | 7.51 | 36 | 11 | -39 |
| Cluster 61 (**23 vox**) |  |  |  |  |  |
|  | R Middle Frontal Gyrus | 7.04 | 29 | 18 | 52 |
| Cluster 62 (**23 vox**) |  |  |  |  |  |
|  | L Superior Medial Gyrus | 8.71 | -8 | 32 | 31 |
| Cluster 63 **(20 vox**) |  |  |  |  |  |
|  | L Superior Frontal Gyrus | 8.01 | -29 | 54 | 24 |
| Cluster 64 (**19 vox**) |  |  |  |  |  |
|  | L Superior Frontal Gyrus | 7.08 | -30 | 53 | 0 |
| Cluster 65 (**19 vox**) |  |  |  |  |  |
|  | R Cerebelum (IV-V) | 6.70 | 9 | -52 | -8 |
| Cluster 66 (**18 vox**) |  |  |  |  |  |
|  | L Middle Temporal Gyrus | 6.90 | -56 | -67 | 1 |
|  | L Middle Temporal Gyrus | 5.28 | -51 | -70 | 1 |
| Cluster 67 (**16 vox**) |  |  |  |  |  |
|  | R Precuneus | 7.43 | 12 | -70 | 63 |
| Cluster 68 (**16 vox**) |  |  |  |  |  |
|  | R Middle Temporal Gyrus | 7.36 | 62 | -54 | 18 |
| Cluster 69 (**16 vox**) |  |  |  |  |  |
|  | Cerebellar Vermis (3) | 6.05 | 6 | -46 | -15 |
|  | R Cerebelum (IV-V) | 5.31 | 8 | -48 | -20 |
| Cluster 70 (**15 vox**) |  |  |  |  |  |
|  | L Insula Lobe | 7.21 | -38 | 2 | 6 |
| Cluster 71 (**15 vox**) |  |  |  |  |  |
|  | R IFG (p. Orbitalis) | 8.29 | 48 | 27 | -5 |
| Cluster 72 (**14 vox**) |  |  |  |  |  |
|  | R Middle Frontal Gyrus | 6.56 | 41 | 6 | 52 |
|  | R Middle Frontal Gyrus | 5.64 | 42 | 2 | 54 |
| Cluster 73 (**14 vox**) |  |  |  |  |  |
|  | R IFG (p. Triangularis) | 7.01 | 54 | 39 | -2 |
| Cluster 74 (**14 vox**) |  |  |  |  |  |
|  | L Inferior Temporal Gyrus | 6.83 | -57 | -63 | -5 |
| Cluster 75 (**13 vox**) |  |  |  |  |  |
|  | R Precuneus | 6.11 | 12 | -63 | 27 |
| Cluster 76 (**12 vox**) |  |  |  |  |  |
|  | R Angular Gyrus | 6.51 | 44 | -60 | 52 |
|  | R Angular Gyrus | 5.68 | 44 | -63 | 49 |
| Cluster 77 **(12 vox**) |  |  |  |  |  |
|  | R Middle Frontal Gyrus | 8.58 | 30 | 51 | 27 |
| Cluster 78 (**12 vox**) |  |  |  |  |  |
|  | L Calcarine Gyrus | 6.40 | -11 | -58 | 10 |
| Cluster 79 (**12 vox**) |  |  |  |  |  |
|  | R Middle Frontal Gyrus | 6.26 | 48 | 47 | 6 |
| Cluster 80 (**12 vox**) |  |  |  |  |  |
|  | R Inferior Temporal Gyrus | 6.40 | 51 | -43 | -18 |
| Cluster 81 (**12 vox**) |  |  |  |  |  |
|  | L Inferior Temporal Gyrus | 9.46 | -45 | 5 | -35 |
| Cluster 82 (**12 vox**) |  |  |  |  |  |
|  | R Cerebelum (IX) | 6.35 | 8 | -46 | -36 |
| Cluster 83 (**11 vox**) |  |  |  |  |  |
|  | R Middle Frontal Gyrus | 6.81 | 41 | 11 | 37 |
| Cluster 84 (**11 vox**) |  |  |  |  |  |
|  | L Superior Medial Gyrus | 7.42 | -5 | 39 | 31 |
| Cluster 85 (**11 vox**) |  |  |  |  |  |
|  | R Superior Occipital Gyrus | 6.32 | 27 | -78 | 28 |
| Cluster 86 (**11 vox**) |  |  |  |  |  |
|  | L SupraMarginal Gyrus | 6.43 | -56 | -25 | 22 |
| Cluster 87 (**11 vox)** |  |  |  |  |  |
|  | L Superior Temporal Gyrus | 6.32 | -60 | -15 | 6 |
| Cluster 88 (**11 vox**) |  |  |  |  |  |
|  | L Middle Occipital Gyrus | 7.55 | -38 | -85 | 6 |
| Cluster 89 (**10 vox**) |  |  |  |  |  |
|  | L Precentral Gyrus | 5.64 | -33 | -6 | 57 |
| Cluster 90 (**10 vox**) |  |  |  |  |  |
|  | R Superior Frontal Gyrus | 7.43 | 20 | 45 | 37 |
| Cluster 91 (**10 vox**) |  |  |  |  |  |
|  | L IFG (p. Opercularis) | 5.60 | -53 | 9 | 7 |
