# Supplementary material for: Volumetric Brain Changes in Older Fallers: A Voxel-Based Morphometric Study
Source: Front Bioeng Biotechnol. 2021 Mar 10;9:610426. doi: 10.3389/fbioe.2021.610426 (PMC7987921; doi:10.3389/fbioe.2021.610426)
Supplement: Supplementary file 4 [file Table_4.docx]

**Appendix 4. Detailed results of VBM analysis according to anatomic toolbox2.2c after adjustment for potential confounders: *t*-test corresponding to the hypothesis that MCI exhibited greater gray matter subvolumes than people with dementia. A threshold of P<0.05, corrected for multiple comparisons based on the false discovery rate (FDR), was applied to the resulting statistical parametric maps. Only clusters with a minimum extent of 10 contiguous voxels are reported.**

|  | **Brain region** | ***t-*score** | **MNI coordinates** | | |
| --- | --- | --- | --- | --- | --- |
| Cluster 1 (**43666 vox**) |  |  |  |  |  |
|  | L Amygdala | 6.96 | -23 | -7 | -14 |
|  | L Hippocampus | 6.90 | -26 | -9 | -17 |
|  | L Amygdala | 6.61 | -20 | -1 | -24 |
|  | R Hippocampus | 6.61 | 26 | -9 | -15 |
|  | L Hippocampus | 5.78 | -30 | -15 | -14 |
|  | L Insula Lobe | 5.59 | -36 | 21 | 6 |
|  | L Fusiform Gyrus | 5.36 | -30 | -34 | -21 |
|  | L Fusiform Gyrus | 5.21 | -30 | -28 | -24 |
|  | L Insula Lobe | 5.11 | -41 | 11 | 0 |
|  | L Rectal Gyrus | 5.04 | -8 | 30 | -23 |
| Cluster 2 (**4860 vox**) |  |  |  |  |  |
|  | L Postcentral Gyrus | 4.36 | -54 | -12 | 37 |
|  | L Postcentral Gyrus | 4.22 | -47 | -22 | 58 |
|  | L Inferior Parietal Lobule | 4.18 | -48 | -52 | 49 |
|  | L Angular Gyrus | 4.16 | -45 | -63 | 43 |
|  | L Postcentral Gyrus | 4.15 | -51 | -13 | 42 |
|  | L Angular Gyrus | 4.14 | -48 | -66 | 39 |
|  | L Inferior Parietal Lobule | 4.10 | -50 | -42 | 48 |
|  | L Inferior Parietal Lobule | 4.04 | -51 | -40 | 46 |
|  | L Postcentral Gyrus | 3.94 | -54 | -10 | 31 |
|  | L Angular Gyrus | 3.92 | -48 | -67 | 28 |
|  | L Angular Gyrus | 3.90 | -42 | -66 | 48 |
| Cluster 3 (**2957 vox**) |  |  |  |  |  |
|  | R Middle Frontal Gyrus | 4.59 | 42 | 53 | 18 |
|  | R Superior Orbital Gyrus | 4.55 | 27 | 36 | -14 |
|  | R Middle Orbital Gyrus | 4.41 | 33 | 53 | -14 |
|  | R Middle Frontal Gyrus | 4.18 | 39 | 57 | 1 |
|  | R Middle Frontal Gyrus | 3.87 | 29 | 57 | 6 |
|  | R Superior Orbital Gyrus | 3.83 | 15 | 63 | -12 |
|  | R Middle Orbital Gyrus | 3.80 | 29 | 54 | -17 |
|  | R IFG (p. Triangularis) | 3.69 | 54 | 39 | -2 |
|  | R IFG (p. Triangularis) | 3.62 | 51 | 36 | 16 |
|  | R IFG (p. Triangularis) | 3.62 | 54 | 38 | 6 |
|  | R Middle Frontal Gyrus | 3.60 | 36 | 53 | 6 |
| Cluster 4 (**877 vox**) |  |  |  |  |  |
|  | L Middle Frontal Gyrus | 4.37 | -36 | 41 | 36 |
|  | L Middle Frontal Gyrus | 4.01 | -24 | 20 | 48 |
|  | L Middle Frontal Gyrus | 3.92 | -32 | 12 | 54 |
|  | L Middle Frontal Gyrus | 3.78 | -21 | 26 | 46 |
|  | L Middle Frontal Gyrus | 3.16 | -32 | 32 | 43 |
|  | L Middle Frontal Gyrus | 3.00 | -24 | 48 | 28 |
|  | L Middle Frontal Gyrus | 2.98 | -39 | 32 | 34 |
|  | L Superior Frontal Gyrus | 2.94 | -21 | 14 | 48 |
|  | L Middle Frontal Gyrus | 2.92 | -39 | 29 | 39 |
|  | L Middle Frontal Gyrus | 2.90 | -30 | 21 | 49 |
|  | L Middle Frontal Gyrus | 2.87 | -35 | 30 | 39 |
| Cluster 5 (**785 vox**) |  |  |  |  |  |
|  | R Middle Frontal Gyrus | 3.62 | 29 | 33 | 45 |
|  | R Middle Frontal Gyrus | 3.56 | 29 | 18 | 52 |
|  | R Middle Frontal Gyrus | 3.45 | 23 | 29 | 43 |
|  | R Superior Frontal Gyrus | 3.42 | 23 | 21 | 48 |
|  | R Middle Frontal Gyrus | 3.41 | 35 | 32 | 43 |
|  | R Middle Frontal Gyrus | 3.25 | 30 | 26 | 46 |
|  | R Middle Frontal Gyrus | 2.91 | 39 | 27 | 43 |
|  | R Middle Frontal Gyrus | 2.90 | 38 | 26 | 49 |
|  | R Middle Frontal Gyrus | 2.79 | 36 | 17 | 52 |
|  | R Middle Frontal Gyrus | 2.68 | 44 | 26 | 42 |
|  | R Superior Frontal Gyrus | 2.55 | 20 | 15 | 52 |
| Cluster 6 (**250 vox**) |  |  |  |  |  |
|  | L Superior Parietal Lobule | 4.26 | -23 | -48 | 70 |
|  | L Superior Parietal Lobule | 3.65 | -30 | -52 | 66 |
|  | L Superior Parietal Lobule | 3.60 | -30 | -48 | 66 |
|  | L Precuneus | 3.13 | -17 | -45 | 69 |
|  | L Postcentral Gyrus | 2.91 | -27 | -40 | 61 |
|  | L Postcentral Gyrus | 2.85 | -23 | -43 | 66 |
| Cluster 7 (**195 vox**) |  |  |  |  |  |
|  | L Inferior Occipital Gyrus | 3.61 | -50 | -73 | -17 |
|  | L Inferior Occipital Gyrus | 2.98 | -45 | -75 | -14 |
|  | L Inferior Occipital Gyrus | 2.94 | -50 | -73 | -8 |
| Cluster 8 (**185 vox**) |  |  |  |  |  |
|  | R IFG (p. Opercularis) | 3.32 | 41 | 12 | 37 |
|  | R IFG (p. Opercularis) | 3.14 | 39 | 11 | 27 |
|  | R IFG (p. Opercularis) | 2.96 | 38 | 14 | 30 |
|  | R IFG (p. Opercularis) | 2.94 | 44 | 11 | 34 |
|  | R IFG (p. Opercularis) | 2.69 | 45 | 14 | 31 |
| Cluster 9 (**161 vox**) |  |  |  |  |  |
|  | L Posterior-Medial Frontal | 4.01 | -5 | -10 | 52 |
|  | L Posterior-Medial Frontal | 3.52 | -2 | -12 | 55 |
|  | R Posterior-Medial Frontal | 2.93 | 2 | -13 | 55 |
| Cluster 10 (**151 vox**) |  |  |  |  |  |
|  | R Middle Temporal Gyrus | 3.04 | 45 | -72 | 21 |
|  | R Middle Occipital Gyrus | 2.85 | 44 | -73 | 28 |
|  | R Middle Occipital Gyrus | 2.63 | 51 | -69 | 28 |
|  | R Angular Gyrus | 2.62 | 44 | -72 | 34 |
|  | R Angular Gyrus | 2.62 | 45 | -70 | 33 |
|  | R Middle Occipital Gyrus | 2.55 | 39 | -76 | 31 |
| Cluster 11 (**111 vox**) |  |  |  |  |  |
|  | L Postcentral Gyrus | 3.13 | -33 | -36 | 54 |
|  | L Postcentral Gyrus | 2.59 | -39 | -34 | 54 |
|  | L Postcentral Gyrus | 2.47 | -35 | -34 | 43 |
|  | L Postcentral Gyrus | 2.37 | -32 | -37 | 46 |
| Cluster 12 (**100 vox**) |  |  |  |  |  |
|  | R Superior Frontal Gyrus | 3.76 | 20 | 45 | 37 |
|  | R Middle Frontal Gyrus | 2.91 | 24 | 42 | 30 |
|  | R Middle Frontal Gyrus | 2.44 | 26 | 36 | 34 |
| Cluster 13 (**95 vox**) |  |  |  |  |  |
|  | R Middle Temporal Gyrus | 3.31 | 51 | -63 | 4 |
|  | R Middle Temporal Gyrus | 3.28 | 48 | -66 | 4 |
|  | R Middle Temporal Gyrus | 2.54 | 53 | -67 | 3 |
| Cluster 14 (**81 vox**) |  |  |  |  |  |
|  | L Precuneus | 3.33 | -9 | -39 | 60 |
| Cluster 15 (**81 vox**) |  |  |  |  |  |
|  | L Superior Frontal Gyrus | 2.87 | -14 | 68 | 9 |
|  | L Superior Frontal Gyrus | 2.86 | -17 | 60 | 13 |
|  | L Superior Medial Gyrus | 2.73 | -9 | 62 | 12 |
|  | L Superior Medial Gyrus | 2.71 | -8 | 63 | 10 |
| Cluster 16 (**69 vox**) |  |  |  |  |  |
|  | L Superior Temporal Gyrus | 2.92 | -53 | -24 | 7 |
| Cluster 17 (**64 vox**) |  |  |  |  |  |
|  | R Posterior-Medial Frontal | 3.69 | 9 | 14 | 46 |
|  | R Superior Medial Gyrus | 2.69 | 8 | 20 | 43 |
| Cluster 18 (**61 vox**) |  |  |  |  |  |
|  | L Middle Temporal Gyrus | 3.02 | -45 | -54 | 15 |
| Cluster 19 (**60 vox**) |  |  |  |  |  |
|  | R ACC | 3.03 | 6 | 47 | 7 |
|  | R Superior Medial Gyrus | 2.73 | 9 | 50 | 4 |
| Cluster 20 (**56 vox**) |  |  |  |  |  |
|  | R Precuneus | 3.38 | 12 | -70 | 63 |
| Cluster 21 (**55 vox**) |  |  |  |  |  |
|  | R Middle Frontal Gyrus | 4.06 | 30 | 51 | 27 |
|  | R Superior Frontal Gyrus | 2.66 | 24 | 51 | 19 |
|  | R Middle Frontal Gyrus | 2.60 | 26 | 54 | 24 |
| Cluster 22 (**54 vox**) |  |  |  |  |  |
|  | R Inferior Temporal Gyrus | 2.85 | 41 | -6 | -47 |
| Cluster 23 (**47 vox**) |  |  |  |  |  |
|  | R Calcarine Gyrus | 2.74 | 5 | -61 | 18 |
|  | R Calcarine Gyrus | 2.64 | 11 | -63 | 10 |
|  | R Calcarine Gyrus | 2.64 | 9 | -61 | 12 |
| Cluster 24 (**46 vox**) |  |  |  |  |  |
|  | R Fusiform Gyrus | 3.40 | 26 | -81 | -12 |
|  | R Fusiform Gyrus | 2.59 | 24 | -73 | -12 |
| Cluster 25 (**45 vox**) |  |  |  |  |  |
|  | R Thalamus | 2.90 | 9 | -9 | 10 |
|  | R Thalamus | 2.60 | 8 | -12 | 7 |
|  | R Thalamus | 2.47 | 9 | -18 | 3 |
|  | R Thalamus | 2.46 | 8 | -15 | 4 |
| Cluster 26 (**44 vox**) |  |  |  |  |  |
|  | R Middle Temporal Gyrus | 3.05 | 50 | -75 | 12 |
| Cluster 27 (**41 vox**) |  |  |  |  |  |
|  | R IFG (p. Opercularis) | 3.00 | 54 | 8 | 28 |
|  | R IFG (p. Opercularis) | 2.55 | 51 | 12 | 30 |
| Cluster 28 (**38 vox**) |  |  |  |  |  |
|  | RPrecentral Gyrus | 3.25 | 54 | 6 | 39 |
| Cluster 29 (**36 vox**) |  |  |  |  |  |
|  | Cerebellar Vermis (4/5) | 2.81 | 0 | -63 | -8 |
| Cluster 30 (**36 vox**) |  |  |  |  |  |
|  | R Inferior Temporal Gyrus | 2.73 | 51 | -43 | -20 |
| Cluster 31 (**36 vox**) |  |  |  |  |  |
|  | R Medial Temporal Pole | 3.48 | 36 | 11 | -39 |
| Cluster 32 (**35 vox**) |  |  |  |  |  |
|  | R Lingual Gyrus | 3.00 | 11 | -52 | 4 |
| Cluster 33 (**34 vox**) |  |  |  |  |  |
|  | L Inferior Temporal Gyrus | 2.70 | -48 | -61 | -11 |
| Cluster 34 (**31 vox**) |  |  |  |  |  |
|  | R Middle Frontal Gyrus | 2.97 | 33 | 35 | 33 |
| Cluster 35 (**29 vox**) |  |  |  |  |  |
|  | R Superior Parietal Lobule | 3.05 | 20 | -49 | 74 |
| Cluster 36 (**28 vox**) |  |  |  |  |  |
|  | R Inferior Temporal Gyrus | 2.82 | 59 | -60 | -12 |
| Cluster 37 (**25 vox**) |  |  |  |  |  |
|  | R Rolandic Operculum | 2.96 | 59 | 9 | 3 |
| Cluster 38 (**24 vox**) |  |  |  |  |  |
|  | L Precentral Gyrus | 3.06 | -39 | 0 | 51 |
|  | L Precentral Gyrus | 2.45 | -44 | 5 | 49 |
| Cluster 39 (**23 vox**) |  |  |  |  |  |
|  | L Middle Occipital Gyrus | 2.79 | -41 | -72 | 1 |
| Cluster 40 (**22 vox**) |  |  |  |  |  |
|  | R Fusiform Gyrus | 2.70 | 39 | -60 | -14 |
| Cluster 41 (**20 vox**) |  |  |  |  |  |
|  | L Middle Frontal Gyrus | 2.92 | -39 | 18 | 48 |
| Cluster 42 (**19 vox**) |  |  |  |  |  |
|  | R Postcentral Gyrus | 2.72 | 36 | -34 | 60 |
|  | R Postcentral Gyrus | 2.54 | 39 | -34 | 66 |
| Cluster 43 (**19 vox**) |  |  |  |  |  |
|  | R Precuneus | 2.78 | 12 | -63 | 27 |
| Cluster 44 (**18 vox**) |  |  |  |  |  |
|  | R Inferior Temporal Gyrus | 2.84 | 60 | -57 | -3 |
| Cluster 45 (**15 vox**) |  |  |  |  |  |
|  | L Posterior-Medial Frontal | 2.54 | -6 | 5 | 63 |
|  | L Posterior-Medial Frontal | 2.45 | -3 | 9 | 57 |
| Cluster 46 (**15 vox**) |  |  |  |  |  |
|  | Cerebellar Vermis (4/5) | 2.74 | -2 | -57 | -2 |
| Cluster 47 (**14 vox**) |  |  |  |  |  |
|  | L IFG (p. Triangularis) | 2.73 | -44 | 44 | 12 |
| Cluster 48 (**14 vox**) |  |  |  |  |  |
|  | L Middle Occipital Gyrus | 2.90 | -38 | -90 | -6 |
| Cluster 49 (**14 vox**) |  |  |  |  |  |
|  | R Insula Lobe | 2.48 | 27 | 23 | -15 |
|  | R Insula Lobe | 2.47 | 32 | 23 | -20 |
|  | R IFG (p. Orbitalis) | 2.43 | 36 | 24 | -18 |
| Cluster 50 (**13 vox**) |  |  |  |  |  |
|  | RPrecentral Gyrus | 3.80 | 41 | -27 | 66 |
|  | RPrecentral Gyrus | 2.63 | 42 | -24 | 63 |
| Cluster 51 (**13 vox**) |  |  |  |  |  |
|  | L Superior Frontal Gyrus | 2.96 | -14 | 50 | 31 |
| Cluster 52 (**13 vox**) |  |  |  |  |  |
|  | R Thalamus | 2.98 | 15 | -24 | 1 |
| Cluster 53 (**13 vox**) |  |  |  |  |  |
|  | R Superior Temporal Gyrus | 2.98 | 53 | -7 | -2 |
| Cluster 54 (**13 vox**) |  |  |  |  |  |
|  | R Inferior Temporal Gyrus | 2.48 | 47 | -51 | -12 |
| Cluster 55 (**12 vox**) |  |  |  |  |  |
|  | R Precuneus | 2.48 | 5 | -57 | 48 |
| Cluster 56 (**11 vox**) |  |  |  |  |  |
|  | R MCC | 2.69 | 8 | 2 | 40 |
| Cluster 57 (**11 vox**) |  |  |  |  |  |
|  | R MCC | 2.72 | 6 | -46 | 37 |
