# Supplementary material for: Volumetric Brain Changes in Older Fallers: A Voxel-Based Morphometric Study
Source: Front Bioeng Biotechnol. 2021 Mar 10;9:610426. doi: 10.3389/fbioe.2021.610426 (PMC7987921; doi:10.3389/fbioe.2021.610426)
Supplement: Supplementary file 5 [file Table_5.docx]

**Appendix 5. Detailed results of VBM analysis according to anatomic toolbox2.2c after adjustment for potential confounders: *t*-test corresponding to the hypothesis that cognitively healthy individuals exhibited greater gray matter subvolumes than people with dementia. A threshold of P<0.05, corrected for multiple comparisons based on the false discovery rate (FDR), was applied to the resulting statistical parametric maps. Only clusters with a minimum extent of 10 contiguous voxels are reported.**

|  | **Brain region** | ***t-*score** | **MNI coordinates** | | |
| --- | --- | --- | --- | --- | --- |
| Cluster 1 (**78015 vox**) |  |  |  |  |  |
|  | L Hippocampus | 7.23 | -26 | -9 | -17 |
|  | R Hippocampus | 6.70 | 26 | -9 | -15 |
|  | L Middle Temporal Gyrus | 6.33 | -59 | -30 | -5 |
|  | L Fusiform Gyrus | 5.78 | -30 | -37 | -20 |
|  | L ParaHippocampal Gyrus | 5.61 | -32 | -27 | -17 |
|  | L ParaHippocampal Gyrus | 5.60 | -32 | -22 | -20 |
|  | L Hippocampus | 5.59 | -30 | -16 | -14 |
|  | L Putamen | 5.57 | -9 | 8 | -9 |
|  | L Insula Lobe | 5.50 | -42 | 11 | 0 |
|  | R Hippocampus | 5.49 | 30 | -30 | -9 |
| Cluster 2 (**1725 vox**) |  |  |  |  |  |
|  | R Crebelum (IX) | 3.84 | 3 | -52 | -59 |
|  | L Cerebelum (IX) | 3.79 | -6 | -52 | -59 |
|  | L Cerebelum (IX) | 3.78 | -9 | -48 | -62 |
|  | L Cerebelum (IX) | 3.64 | -8 | -51 | -54 |
|  | R Cerebelum (IX) | 3.45 | 9 | -51 | -51 |
|  | L Cerebelum (IX) | 3.33 | -8 | -46 | -51 |
|  | L Cerebelum (IX) | 3.17 | 0 | -52 | -45 |
|  | R Cerebelum (IX) | 2.93 | 5 | -45 | -38 |
|  | R Cerebelum (IX) | 2.93 | 9 | -61 | -50 |
|  | R Cerebelum (IX) | 2.90 | 8 | -60 | -48 |
| Cluster 3 (**964 vox**) |  |  |  |  |  |
|  | R Superior Frontal Gyrus | 3.68 | 24 | -6 | 57 |
|  | R Superior Frontal Gyrus | 3.52 | 26 | -1 | 61 |
|  | R Superior Frontal Gyrus | 3.47 | 29 | 2 | 58 |
|  | R Superior Frontal Gyrus | 3.19 | 21 | 14 | 54 |
|  | R Superior Frontal Gyrus | 3.13 | 21 | -4 | 69 |
|  | RPrecentral Gyrus | 3.04 | 47 | 6 | 48 |
|  | R Middle Frontal Gyrus | 3.03 | 42 | 8 | 51 |
|  | R Middle Frontal Gyrus | 3.02 | 45 | 0 | 52 |
|  | R Middle Frontal Gyrus | 2.97 | 39 | 3 | 55 |
|  | RPrecentral Gyrus | 2.60 | 38 | 2 | 51 |
|  | R Middle Frontal Gyrus | 2.52 | 41 | 9 | 60 |
| Cluster 4 (**670 vox**) |  |  |  |  |  |
|  | R Middle Frontal Gyrus | 3.54 | 30 | 18 | 54 |
|  | R Middle Frontal Gyrus | 3.50 | 36 | 32 | 43 |
|  | R Middle Frontal Gyrus | 3.45 | 29 | 29 | 46 |
|  | R Middle Frontal Gyrus | 3.34 | 29 | 33 | 45 |
|  | R Middle Frontal Gyrus | 3.21 | 23 | 29 | 43 |
|  | R Superior Frontal Gyrus | 3.10 | 21 | 29 | 51 |
|  | R Superior Frontal Gyrus | 2.77 | 23 | 23 | 52 |
|  | R Middle Frontal Gyrus | 2.67 | 35 | 23 | 57 |
|  | R Middle Frontal Gyrus | STAT = 2.21 | 38 | 17 | 51 |
| Cluster 5 (**474 vox**) |  |  |  |  |  |
|  | L Superior Parietal Lobule | 4.16 | -23 | -48 | 70 |
|  | L Precuneus | 3.45 | -17 | -45 | 69 |
|  | L Superior Parietal Lobule | 3.07 | -30 | -46 | 64 |
|  | L Postcentral Gyrus | 2.97 | -36 | -36 | 51 |
|  | L Postcentral Gyrus | 2.92 | -36 | -36 | 63 |
|  | L Postcentral Gyrus | 2.89 | -30 | -42 | 63 |
|  | L Postcentral Gyrus | 2.68 | -27 | -37 | 69 |
|  | L Superior Parietal Lobule | 2.62 | -24 | -54 | 69 |
|  | L Superior Parietal Lobule | 2.62 | -20 | -54 | 69 |
|  | L Postcentral Gyrus | 2.61 | -41 | -34 | 57 |
|  | L Postcentral Gyrus | 2.59 | -39 | -34 | 67 |
| Cluster 6 (**365 vox**) |  |  |  |  |  |
|  | L Middle Frontal Gyrus | 3.09 | -36 | 8 | 63 |
|  | L Middle Frontal Gyrus | 2.99 | -32 | 12 | 55 |
|  | L Middle Frontal Gyrus | 2.98 | -23 | 26 | 49 |
|  | L Superior Frontal Gyrus | 2.97 | -20 | 30 | 48 |
|  | L Middle Frontal Gyrus | 2.79 | -36 | 14 | 58 |
|  | L Superior Frontal Gyrus | 2.74 | -20 | 32 | 40 |
|  | L Middle Frontal Gyrus | 2.70 | -26 | 20 | 51 |
|  | L Middle Frontal Gyrus | 2.60 | -26 | 26 | 39 |
|  | L Precentral Gyrus | 2.18 | -32 | 0 | 60 |
| Cluster 7 (**344 vox**) |  |  |  |  |  |
|  | R IFG (p. Triangularis) | 3.92 | 54 | 29 | 12 |
|  | R IFG (p. Triangularis) | 3.63 | 51 | 35 | 15 |
|  | R IFG (p. Triangularis) | 3.07 | 53 | 36 | 10 |
|  | R IFG (p. Triangularis) | 3.07 | 56 | 35 | 10 |
|  | R IFG (p. Triangularis) | 2.80 | 50 | 30 | 1 |
|  | R IFG (p. Triangularis) | 2.67 | 44 | 30 | 16 |
|  | R IFG (p. Triangularis) | 2.60 | 44 | 39 | 9 |
|  | R Middle Frontal Gyrus | 2.38 | 42 | 39 | 13 |
|  | R IFG (p. Triangularis) | 2.33 | 56 | 32 | 1 |
| Cluster 8 (**275 vox**) |  |  |  |  |  |
|  | L Middle Occipital Gyrus | 3.24 | -29 | -66 | 39 |
|  | L Superior Parietal Lobule | 3.08 | -23 | -72 | 43 |
|  | L Superior Occipital Gyrus | 2.85 | -26 | -72 | 36 |
|  | L Middle Occipital Gyrus | 2.70 | -27 | -70 | 30 |
|  | L Superior Parietal Lobule | 2.55 | -21 | -66 | 45 |
|  | L Middle Occipital Gyrus | 2.47 | -29 | -79 | 36 |
| Cluster 9 (**244 vox**) |  |  |  |  |  |
|  | L Precentral Gyrus | 2.94 | -39 | -9 | 58 |
|  | L Precentral Gyrus | 2.84 | -30 | -9 | 63 |
|  | L Precentral Gyrus | 2.75 | -27 | -6 | 55 |
|  | L Precentral Gyrus | 2.57 | -35 | -10 | 55 |
|  | L Precentral Gyrus | 2.54 | -44 | -15 | 60 |
|  | L Precentral Gyrus | 2.49 | -33 | -6 | 55 |
| Cluster 10 (**163 vox**) |  |  |  |  |  |
|  | R Middle Frontal Gyrus | 3.53 | 30 | 51 | 27 |
|  | R Middle Frontal Gyrus | 3.29 | 24 | 45 | 31 |
|  | R Superior Frontal Gyrus | 2.87 | 24 | 44 | 37 |
|  | R Middle Frontal Gyrus | 2.80 | 30 | 47 | 30 |
|  | R Superior Frontal Gyrus | 2.49 | 23 | 36 | 33 |
| Cluster 11 (**129 vox**) |  |  |  |  |  |
|  | L Precuneus | 3.44 | -9 | -39 | 60 |
|  | L Posterior-Medial Frontal | 2.95 | -5 | -22 | 52 |
|  | L Paracentral Lobule | 2.42 | -3 | -30 | 54 |
|  | L Paracentral Lobule | 2.39 | -5 | -28 | 52 |
| Cluster 12 (**91 vox**) |  |  |  |  |  |
|  | R Superior Parietal Lobule | 3.09 | 20 | -49 | 74 |
|  | R Precuneus | 2.94 | 9 | -54 | 73 |
|  | R Postcentral Gyrus | 2.91 | 15 | -54 | 72 |
|  | R Precuneus | 2.82 | 12 | -57 | 71 |
|  | R Precuneus | 2.77 | 5 | -52 | 69 |
|  | R Precuneus | 2.64 | 6 | -55 | 70 |
| Cluster 13 (**84 vox**) |  |  |  |  |  |
|  | L Precuneus | 2.47 | -6 | -55 | 54 |
|  | L Precuneus | 2.46 | -9 | -51 | 54 |
| Cluster 14 (**83 vox**) |  |  |  |  |  |
|  | L Middle Frontal Gyrus | 2.80 | -41 | 20 | 49 |
|  | L Middle Frontal Gyrus | 2.45 | -32 | 21 | 49 |
| Cluster 15 (**70 vox**) |  |  |  |  |  |
|  | R Posterior-Medial Frontal | 2.75 | 9 | 14 | 46 |
|  | R MCC | 2.62 | 8 | 23 | 37 |
|  | R Posterior-Medial Frontal | 2.47 | 5 | 17 | 46 |
|  | R Superior Medial Gyrus | 2.46 | 8 | 20 | 42 |
| Cluster 16 (**49 vox**) |  |  |  |  |  |
|  | R Precuneus | 3.02 | 5 | -46 | 57 |
| Cluster 17 (**42 vox**) |  |  |  |  |  |
|  | Cerebellar Vermis (7) | 2.98 | 0 | -78 | -30 |
| Cluster 18 (**40 vox**) |  |  |  |  |  |
|  | R Superior Medial Gyrus | 2.71 | 5 | 59 | 7 |
| Cluster 19 (**32 vox**) |  |  |  |  |  |
|  | R Paracentral Lobule | 2.88 | 9 | -42 | 69 |
|  | R Paracentral Lobule | 2.40 | 8 | -42 | 61 |
| Cluster 20 (**30 vox**) |  |  |  |  |  |
|  | R Rectal Gyrus | 2.83 | 2 | 57 | -23 |
| Cluster 21 (**30 vox**) |  |  |  |  |  |
|  | L Inferior Temporal Gyrus | 2.49 | -36 | 8 | -42 |
| Cluster 22 (**28 vox**) |  |  |  |  |  |
|  | R Inferior Parietal Lobule | 2.45 | 39 | -46 | 40 |
|  | R Inferior Parietal Lobule | 2.37 | 33 | -42 | 46 |
|  | R Inferior Parietal Lobule | 2.29 | 36 | -43 | 43 |
| Cluster 23 (**26 vox**) |  |  |  |  |  |
|  | R Lingual Gyrus | 2.96 | 11 | -72 | -6 |
|  | R Lingual Gyrus | 2.38 | 8 | -67 | -8 |
| Cluster 24 (**23 vox**) |  |  |  |  |  |
|  | R Middle Frontal Gyrus | 2.85 | 35 | 36 | 34 |
| Cluster 25 (**22 vox**) |  |  |  |  |  |
|  | L Cuneus | 2.63 | -14 | -79 | 36 |
|  | L Superior Occipital Gyrus | 2.22 | -17 | -78 | 40 |
| Cluster 26 (**21 vox**) |  |  |  |  |  |
|  | L Superior Occipital Gyrus | 2.74 | -23 | -85 | 25 |
|  | L Middle Occipital Gyrus | 2.44 | -23 | -85 | 21 |
| Cluster 27 (**21 vox**) |  |  |  |  |  |
|  | L Cerebelum (Crus 2) | 2.55 | -23 | -88 | -39 |
| Cluster 28 (**19 vox**) |  |  |  |  |  |
|  | L Superior Parietal Lobule | 2.89 | -24 | -66 | 61 |
| Cluster 29 (**19 vox**) |  |  |  |  |  |
|  | L Superior Frontal Gyrus | 2.69 | -23 | 47 | 40 |
| Cluster 30 (**17 vox**) |  |  |  |  |  |
|  | Cerebellar Vermis (9) | 2.48 | -5 | -52 | -32 |
| Cluster 31 (**16 vox**) |  |  |  |  |  |
|  | L Superior Parietal Lobule | 2.84 | -14 | -72 | 54 |
| Cluster 32 (**16 vox**) |  |  |  |  |  |
|  | R Superior Frontal Gyrus | 2.53 | 14 | 62 | 22 |
| Cluster 33 (**15 vox**) |  |  |  |  |  |
|  | L Superior Frontal Gyrus | 2.53 | -15 | 41 | 42 |
| Cluster 34 (**14 vox**) |  |  |  |  |  |
|  | R Postcentral Gyrus | 2.88 | 29 | -42 | 65 |
| Cluster 35 (**14 vox**) |  |  |  |  |  |
|  | L Inferior Parietal Lobule | 2.71 | -27 | -45 | 45 |
| Cluster 36 (**14 vox**) |  |  |  |  |  |
|  | R Cerebelum (Crus 1) | 2.73 | 41 | -82 | -35 |
| Cluster 37 (**13 vox**) |  |  |  |  |  |
|  | R Cuneus | 2.44 | 6 | -78 | 27 |
| Cluster 38 (**12 vox**) |  |  |  |  |  |
|  | L Precuneus | 2.54 | -6 | -70 | 59 |
| Cluster 39 (**12 vox**) |  |  |  |  |  |
|  | L Lingual Gyrus | 2.78 | -12 | -90 | -15 |
| Cluster 40 (**11 vox**) |  |  |  |  |  |
|  | R Superior Frontal Gyrus | 3.44 | 32 | -10 | 70 |
| Cluster 41 (**10 vox**) |  |  |  |  |  |
|  | L Superior Parietal Lobule | 2.81 | -12 | -72 | 42 |
| Cluster 42 (**10 vox**) |  |  |  |  |  |
|  | R Middle Frontal Gyrus | 2.69 | 42 | 41 | 31 |
| Cluster 43 (**10 vox**) |  |  |  |  |  |
|  | L IFG (p. Triangularis) | 2.69 | -50 | 27 | 24 |
