# Supplementary material for: Volumetric Brain Changes in Older Fallers: A Voxel-Based Morphometric Study
Source: Front Bioeng Biotechnol. 2021 Mar 10;9:610426. doi: 10.3389/fbioe.2021.610426 (PMC7987921; doi:10.3389/fbioe.2021.610426)
Supplement: Supplementary file 6 [file Table_6.docx]

**Appendix 6. Detailed results of VBM analysis according to anatomic toolbox2.2c after adjustment for potential confounders: *t*-test corresponding to the hypothesis that fallers with dementia exhibited greater gray matter subvolumes than non-fallers with dementia. A threshold of P<0.05, corrected for multiple comparisons based on the false discovery rate (FDR), was applied to the resulting statistical parametric maps. Only clusters with a minimum extent of 10 contiguous voxels are reported.**

| **Cluster size** | **Brain region** | ***t-*score** | **MNI coordinates** | | |
| --- | --- | --- | --- | --- | --- |
| Cluster 1 (**462 vox**) | R Caudate nucleus | 4,54 | -20 | -3 | 22 |
| Cluster 2 (**290 vox**) | L Caudate nucleus | 4,44 | 14 | 0 | 12 |
| Cluster 3 (**177 vox**) | R Caudate nucleus | 5,11 | -14 | -30 | 38 |
| Cluster 4 (**165 vox**) | L Mid Cingulum | 4,86 | 18 | 24 | -3 |
| Cluster 5 (**48 vox**) | L Insula | 3,99 | -16 | 24 | 2 |
| Cluster 6 (**46 vox**) | R Insula | 5,77 | -30 | 6 | 12 |
| Cluster 7 (**39 vox**) | Inf L Temporal | 4,76 | 34 | 8 | 15 |
| Cluster 8 (**26 vox**) | L Supp Motor Area | 3,83 | 18 | -30 | 45 |
| Cluster 9 (**26 vox**) | L Putamen | 4,28 | -8 | 21 | 60 |
| Cluster 10 (**25 vox**) | R Mid Cingulum | 4,34 | -33 | -6 | 10 |
| Cluster 11 (**23 vox**) | R Angular | 4,01 | -50 | -45 | -27 |
| Cluster 12 (**21 vox**) | L Fusiform | 4,26 | -48 | -60 | -18 |
| Cluster 13 (**21 vox**) | R Putamen | 4,05 | -33 | -20 | 2 |
| Cluster 14 (**11 vox**) | L Crus Cerebelum | 3,69 | -60 | -50 | -22 |
| Cluster 15 (**11 vox**) | R Pallidum | 3,95 | 38 | -68 | 42 |
