# Supplementary material for: Volumetric Brain Changes in Older Fallers: A Voxel-Based Morphometric Study
Source: Front Bioeng Biotechnol. 2021 Mar 10;9:610426. doi: 10.3389/fbioe.2021.610426 (PMC7987921; doi:10.3389/fbioe.2021.610426)
Supplement: Supplementary file 7 [file Table_7.docx]

**Appendix 7. Detailed results of VBM analysis according to anatomic toolbox2.2c after adjustment for potential confounders: *t-*test corresponding to the hypothesis that fallers with mild cognitive impairment exhibited greater gray matter subvolumes than non-fallers with mild cognitive impairment. A threshold of P<0.05, corrected for multiple comparisons based on the false discovery rate (FDR), was applied to the resulting statistical parametric maps. Only clusters with a minimum extent of 10 contiguous voxels are reported.**

| **Cluster size** | **Brain region** | ***t-*score** | **MNI coordinates** | | |
| --- | --- | --- | --- | --- | --- |
| Cluster 1 (**193 vox**) | L Caudate nucleus | 5,17 | 22 | -2 | 22 |
| Cluster 2 (**168 vox**) | R Caudate nucleus | 4,67 | -16 | 2 | -15 |
| Cluster 3 (**41 vox**) | L Amygdala | 4,41 | 21 | 8 | -14 |
| Cluster 4 (**36 vox**) | L Olfactory | 4,6 | -24 | 16 | 48 |
